# Functional characterization of proton antiport regulation in the thylakoid membrane

**DOI:** 10.1093/plphys/kiab135

**Published:** 2021-03-20

**Authors:** Michał Uflewski, Sarah Mielke, Viviana Correa Galvis, Thekla von Bismarck, Xiaoheng Chen, Enrico Tietz, Jeremy Ruß, Marcin Luzarowski, Ewelina Sokolowska, Aleksandra Skirycz, Jürgen Eirich, Iris Finkemeier, Mark Aurel Schöttler, Ute Armbruster

**Affiliations:** 1 Max Planck Institute of Molecular Plant Physiology, Potsdam 14476, Germany; 2 Boyce Thompson Institute, Ithaca 14853, New York; 3 Plant Physiology, Institute of Plant Biology and Biotechnology, University of Münster, Münster 48149, Germany

## Abstract

During photosynthesis, energy is transiently stored as an electrochemical proton gradient across the thylakoid membrane. The resulting proton motive force (pmf) is composed of a membrane potential (ΔΨ) and a proton concentration gradient (ΔpH) and powers the synthesis of ATP. Light energy availability for photosynthesis can change very rapidly and frequently in nature. Thylakoid ion transport proteins buffer the effects that light fluctuations have on photosynthesis by adjusting pmf and its composition. Ion channel activities dissipate ΔΨ, thereby reducing charge recombinations within photosystem II. The dissipation of ΔΨ allows for increased accumulation of protons in the thylakoid lumen, generating the signal that activates feedback downregulation of photosynthesis. Proton export from the lumen via the thylakoid K^+^ exchange antiporter 3 (KEA3), instead, decreases the ΔpH fraction of the pmf and thereby reduces the regulatory feedback signal. Here, we reveal that the Arabidopsis (*Arabidopsis thaliana*) KEA3 protein homo-dimerizes via its C-terminal domain. This C-terminus has a regulatory function, which responds to light intensity transients. Plants carrying a C-terminus-less KEA3 variant show reduced feed-back downregulation of photosynthesis and suffer from increased photosystem damage under long-term high light stress. However, during photosynthetic induction in high light, KEA3 deregulation leads to an increase in carbon fixation rates. Together, the data reveal a trade-off between long-term photoprotection and a short-term boost in carbon fixation rates, which is under the control of the KEA3 C-terminus.

## Teaser

The regulatory C-terminus of the thylakoid Kexchange antiporter 3 (KEA3) is required for mitigating high light stress and protein dimerization.

## Introduction

Solar energy is the driving force for nearly all life on Earth. This requires its conversion into metabolic energy by photosynthesis. At the heart of photosynthesis are light-induced charge separations within the photosystems. Plants have evolved a plethora of regulatory mechanisms that buffer fluctuations in solar energy supply and thus avoid charge recombination events within the photosystems, which would otherwise lead to the generation of reactive oxygen species and photo-oxidative damage (Davis et al., 2016; Foyer, 2018). Light fluctuations can be particularly frequent and extreme within the dense canopies of crop fields, which are the fundamental basis for our food and a source of renewable energy (Kaiser et al., 2018). A dawning hunger crisis demands innovative and effective strategies for enhancing crop photosynthesis and yield (Ort et al., 2015; Simkin et al., 2019). Such efforts will greatly benefit from an in-depth understanding of the regulatory buffering network that underpins dynamic photosynthesis.

In the thylakoid membrane of plant chloroplasts, light-powered linear electron transport occurs from water at the lumenal side to NADPH at the stromal side and involves the two photosystems (PSII and PSI) and a series of redox carriers including those of the Cytochrome *b_6_f* (Cyt *b_6_f*) complex. At PSII and Cyt *b_6_f*, these electron transfer reactions are tightly coupled to the accumulation of protons (H^+^) in the thylakoid lumen. Together, this leads to the generation of an electric potential (Δψ) and a concentration gradient (ΔpH) across the thylakoid membrane. Δψ and ΔpH are thermodynamically equivalent and provide the proton motive force (pmf), which drives H^+^-coupled ATP production via the ATP synthase (Mitchell, 1966). ATP and NADPH power the carbon fixation reactions of the Calvin–Benson–Bassham (CBB) cycle.

Aside from its function in ATP synthesis, ΔpH plays a regulatory role for photosynthesis (reviewed by Armbruster et al., 2017). Above a certain concentration threshold, lumenal protons switch on energy-dependent quenching (qE), which is the main component of non-photochemical quenching (NPQ) in plants (Demmig-Adams et al., 1996), and slow down electron transport by decreasing the rates of plastoquinol oxidation at Cyt *b_6_f* (“photosynthetic control”; Tikhonov et al., 1981; Hope et al., 1994; Finazzi, 2002; Takizawa et al., 2007). In angiosperms, qE is activated by protonation of the thylakoid PsbS protein at the lumenal side, which occurs below a lumen pH of 6.8 (Takizawa et al., 2007). Protonated PsbS induces the rearrangement of PSII supercomplexes, a process that switches on the dissipation of absorbed light energy as heat (Li et al., 2000, 2004; Correa Galvis et al., 2016; Sacharz et al., 2017). qE is further sustained by zeaxanthin, produced by the low pH-activated lumenal enzyme violaxanthin de-epoxidase (VDE; Hager, 1969; Niyogi, 1998).

The fraction of pmf that is stored as ΔpH, and thus its regulatory function, can be adjusted by the activity of thylakoid ion transport proteins. Because the proton buffering capacity of the lumen is high, ΔΨ dissipation by passive ion channels is required for storage of pmf as ΔpH (Nicholls and Ferguson, 2002; Kramer et al., 2004). Light-powered proton transfer into the lumen was shown to be accompanied by the passive flux of Cl^−^, Mg^2+^, and potentially K^+^ (Hind et al., 1974). Of all the conceivable counter-ion channels in the thylakoid membrane, only the Cl^−^-flux mediating voltage-dependent Cl^−^ channel 1 (VCCN1) has been characterized on molecular level so far (Duan et al., 2016; Herdean et al., 2016). Lack of VCCN1 in Arabidopsis (*Arabidopsis thaliana*) results in increased light-induced pmf, larger ΔΨ, and a slower induction of NPQ (Duan et al., 2016; Herdean et al., 2016). In plants overexpressing *VCCN1*, an opposite photosynthetic phenotype with lower pmf and storage exclusively as ΔpH was recorded (Herdean et al., 2016). These results support the notion that VCCN1-mediated Cl^−^ influx into the lumen contributes to the dissipation of ΔΨ and thereby accelerates the induction of pH-dependent NPQ. VCCN1 was shown to be voltage-gated and thus responsive to ΔΨ (Herdean et al., 2016). High thylakoid ΔΨ facilitates charge recombination within PSII (Davis et al., 2016) and VCCN1 activity was shown to protect PSII function in dynamic light environments (Duan et al., 2016; Höhner et al., 2019).

Proton/cation antiport via the K^+^ exchange antiporter 3 (KEA3) pushes pmf composition toward higher ΔΨ, while not generally altering the amplitude of light-induced pmf (Armbruster et al., 2014; Kunz et al., 2014; Dukic et al., 2019; Wang and Shikanai, 2019; Correa Galvis et al., 2020). KEA3 exports protons from the lumen in exchange for another cation, most likely K^+^ (Armbruster et al., 2014; Tsujii et al., 2019). While Cl^−^-influx into the lumen via VCCN1 was shown to be required for fast NPQ activation during photosynthetic induction (i.e. after transition from dark to light; [Duan et al., 2016; Herdean et al., 2016]), H^+^-export from the lumen via KEA3 is mandatory for fast NPQ relaxation directly after a sudden drop in light intensity (Armbruster et al., 2014). Arabidopsis *kea3* mutants displayed strongly delayed NPQ relaxation and were negatively affected in their capacity for CO_2_ assimilation after transition to low light (Armbruster et al., 2014). These results identified KEA3 as an obligatory factor for efficient photosynthesis after a drop in light intensity and revealed that during such light transients, H^+^-efflux via the ATP synthase is not sufficient for the rapid downregulation of the lumenal proton concentration and pH-dependent NPQ. Consequently, a physiological relevance for KEA3 under ATP synthase-limited conditions was proposed (Armbruster et al., 2014). Indeed, KEA3 activity was shown to promote photosynthesis and growth of mutants that lack the CONSERVED ONLY IN THE GREEN LINEAGE 160 (CGL160) protein. CGL160 acts as an ATP synthase assembly factor and *cgl160* mutants have only 20% of WT ATP synthase and high pmf (Rühle et al., 2014; Fristedt et al., 2015). *cgl160* plants, which additionally lacked a functional *KEA3* gene, had the same light-induced pmf as *cgl160* single mutants, but a much higher fraction of pmf stored as ΔpH (Correa Galvis et al., 2020). As a result, *cgl160kea3* double mutants displayed strongly increased NPQ as well as decreased Cyt *b_6_f* turnover, together resulting in reduced carbon fixation rates and slower biomass accumulation when compared with *cgl160* single knock-outs.

In previous work, three different mRNA splice forms of the *KEA3* gene were detected in Arabidopsis (Armbruster et al., 2016). The main KEA3 isoform on both transcript and protein levels is *KEA3.2*/KEA3.2 (Armbruster et al., 2016). This isoform consists of a N-terminal transit peptide for chloroplast targeting, a membrane-intrinsic cation/H^+^ antiporter (CPA2) domain, and a conserved K^+^ transport/nucleotide-binding (KTN) regulatory domain at its soluble C-terminus (Armbruster et al., 2014). Arabidopsis plants overexpressing *KEA3.2* exhibited similar NPQ in high light and faster NPQ relaxation when compared with WT after transition to low light (Armbruster et al., 2016). The same was shown for transfected leaf sections of *Nicotiana benthamiana* that expressed Arabidopsis *KEA3.2* as compared to a control (Armbruster et al., 2016). The overexpression of the minor *KEA3.3* isoform in both Arabidopsis and *N. benthamiana* caused reduced NPQ in high light. KEA3.3 completely lacks the soluble C-terminus. Together, these results suggested that KEA3.2 is inhibited in high light and that activity regulation involves the KEA3.2 C-terminus (Armbruster et al., 2016). The third KEA3 isoform that was detected on transcript level, *KEA3.1*, has a truncated C-terminus (Armbruster et al., 2016). Overexpression attempts for *KEA3.1* were unsuccessful, potentially due to a reduced stability of KEA3.1 when compared with the other two isoforms (Armbruster et al., 2016). Expression of low amounts of *KEA3.1* led to a partial complementation of the *kea3-1* photosynthetic phenotypes, suggesting this KEA3 version is functional.

Further support for a light intensity-dependent activity of the main KEA3.2 isoform came from light response curves of plants with different KEA3.2 levels. *kea3* mutants had higher NPQ than WT at low and medium light intensities, while *KEA3.2* overexpressors had lower NPQ (Wang and Shikanai, 2019). In high light, differences in pmf composition and NPQ between plants with different KEA3.2 levels were small (Armbruster et al., 2014, 2016; Wang and Shikanai, 2019). Plants overexpressing the mutated *disturbed proton gradient regulation* (*DPGR*) version of KEA3.2 were additionally analyzed. This DPGR version of KEA3.2 has been derived by EMS mutagenesis and has Gly422 exchanged by Arg (Wang et al., 2017; Wang and Shikanai, 2019). The *oeDPGR* plants showed reduced NPQ particularly in high light, which was accompanied by low pmf and increased membrane conductivity for protons (*g*_H_^+^) and no significant change in pmf composition when compared with plants overexpressing *KEA3.2* (Wang and Shikanai, 2019). These findings were interpreted as the *DPGR* mutation causing changes in the coupling of protons and cations during antiport, with increased amounts of protons being exchanged against K^+^ or proton slippage (Wang and Shikanai, 2019).

In this study, we further characterized the Arabidopsis KEA3 ion transport protein and the activity regulation via its C-terminal domain. For this we (1) mapped the mature N-terminus of KEA3, (2) characterized photosynthesis of *kea3-1* plants that expressed either *KEA3.3-GFP* or *KEA3.2-GFP* from the *KEA3* promotor, and (3) revealed KEA3 protein interactions.

## Results

### Characterization of the mature KEA3 protein in the thylakoid membrane

**Figure 1:**
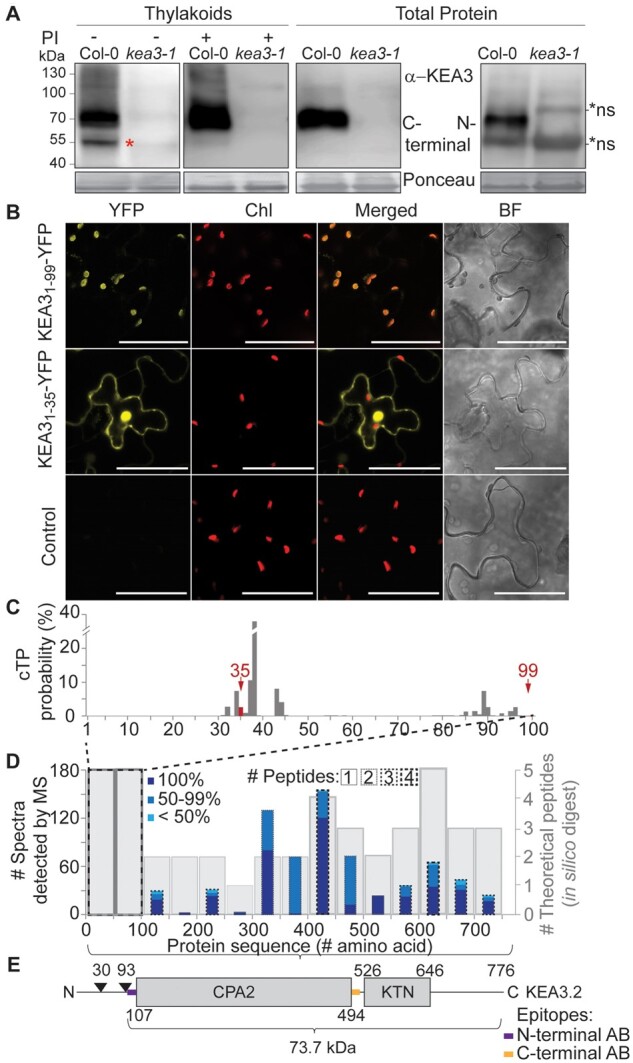
Determination of the mature KEA3 protein sequence. A, Using an antibody recognizing all KEA3 isoforms at the C-terminus, KEA3 is detected in total protein extracts and isolated thylakoids of the Arabidopsis WT Col-0 as one band migrating at around ∼70 kDa. A second lower molecular weight band of KEA3 present in isolated thylakoids corresponds to a degradation product (red asterisk) that is avoided by using protease inhibitors (PIs) during thylakoid isolation. A second KEA3 antibody against a stretch of AA N-terminal of the CPA2 domain also recognized KEA3 as one band at around 70 kDa. Two non-specific (n.s.) bands are marked by black asterisks. Ponceau Red (Ponceau) stain of the membrane prior to immune-detection is shown. B, YFP fused to the first 99 N-terminal AA of KEA3 is targeted to the chloroplast as determined by fluorescence microscopy of transiently expressing *Nicotiana benthamiana* leaf sections. The first 35 N-terminal AA are insufficient for chloroplast targeting (Chl, chlorophyll fluorescence; BF, brightfield; the scale bar represents 50 µm). C, cTP prediction by TargetP 2.0 gives highest probability for cleavage around AA 32–44 peaking at S38. Another probability peak can be observed between AA 85–96 with highest probability for cleavage at Y89. cTP lengths tested in C are marked in red and with arrow. D, No KEA3 peptides can be detected within the first 100 N-terminal AA of KEA3 by MS analysis after tryptic digests of GFP pull-down from thylakoid membranes of *oeKEA3.2-GFP* and *oeKEA3.3-GFP*. Height of bar shows number of spectra detected (left axis), the shade of blue within the bar the confidence levels as calculated by the SEQUEST algorithm, and the stroke and dash of the bar border the number of different peptides that were detected in a given window of 50 AA as indicated by # peptides. The gray bars in the background indicate the number of different possible peptides (right axis) that start within the given window as predicted by digital tryptic digest. E, Linear representation of the main KEA3.2 isoform with arrows at the two previously reported cTP predictions of 30 and 93 AA, the cation proton antiport 2 (CPA2) and K^+^ transport nucleotide binding (KTN) domains, and positions of epitopes used for antibody generation.

While KEA3.2 was shown to be the major KEA3 isoform (Armbruster et al., 2016), it has been discussed that thylakoids also harbor detectable amounts of a smaller KEA3 isoform (Wang et al., 2017). When thylakoid proteins from Col-0, the Arabidopsis WT used in this study, were separated on an SDS-PAGE and detected with a KEA3-specific antibody (Armbruster et al., 2014), the majority of the KEA3 signal was observed around 70 kDa and an additional smaller sized KEA3 band was visible at around 50 kDa (Figure 1, A). However, after using protease inhibitors in all steps of thylakoid isolation, the smaller band was no longer detectable (Figure 1, A). This result strongly suggests that the lower molecular weight band of KEA3 represents a degradation product that occurs during thylakoid isolation in the absence of protease inhibitors. This finding is underlined by the presence of KEA3 as one band of ∼70 kDa in total protein extracts (Figure 1, A). Both, the previously published KEA3 antibody recognizing a region between the CPA2 transport domain and the C-terminal KTN domain (C-terminal antibody, Armbruster et al., 2014), as well as a newly made antibody against the N-terminus of KEA3 upstream of the CPA2 domain (N-terminal antibody), only detected one specific band in total protein extracts (Figure 1, A). The N-terminal antibody, however, was less specific than the C-terminal and yielded two additional bands, one at slightly higher and one at slightly lower molecular weight than KEA3 (Figure 1, A). Thus, in further experiments, the C-terminal antibody was used for KEA3 detection.

Previous reports on KEA3 differed in their prediction of the chloroplast targeting peptide (cTP) cleavage site with either 93 or 30 amino acids (AA) comprising targeting peptides, respectively (Armbruster et al., 2014; Wang et al., 2017). The latter prediction would result in a mature protein with a long N-terminal stretch preceding the CPA2 transport domain. This N-terminal stretch contains one cysteine, which could potentially be involved in redox-regulation of KEA3 activity (Wang et al., 2017). To narrow down the size of the KEA3 cTP, different lengths of the KEA3 N-terminus were fused to YFP and the subcellular location of the fusion product was observed by fluorescence microscopy. While the 99 N-terminal AA of KEA3 resulted in a localization of YFP in the chloroplast (Figure 1, B), the first 35 N-terminal AA were not sufficient to target YFP to this organelle. For the initial prediction of the 93 AA comprising cTP, the TargetP program was used (Armbruster et al., 2014). However, since then the TargetP algorithm has been refined (Almagro Armenteros et al., 2019). The updated algorithm calculates cTP cleavage probabilities for every AA in the N-terminal region of a protein (Figure 1, C). The highest probability for cleavage of the KEA3 cTP is predicted between S38 and S39 and thus downstream of the shorter cTP version of 35 AA that was not sufficient to target YFP to the chloroplast. Another probability peak is around AA 89 (Figure 1, C). To obtain more information on the correct N-terminus of the mature KEA3 protein, we analyzed a data set that we obtained from proteomics analysis of immunoprecipitated KEA3 (Supplemental Figure S1 and Supplemental Data Set S1). From seven independent experiments, we obtained a total number of 717 spectra that were assigned to KEA3 peptides (Supplementary Data Set S1). While tryptic digest should result in 10 peptides deriving from the 100 most N-terminal AAs of KEA3, none of these peptides were detected by the MS analysis (Figure 1, D). The first detected peptide started at AA 126. In the remaining KEA3 protein at least one KEA3 peptide with multiple spectra was detected in a given window of 50 AAs (Figure 1, D). Together, the data suggest that cTP cleavage occurs close to the start of the CPA2 transport domain, but upstream of the epitope sequence that was used to generate the functional N-terminal KEA3 antibody (Figure 1, A), which is located just before the CPA2 domain and spans AA 96-110 (Figure 1, E). Taking the relatively high probability for the cleavage between Y89 and A90 by the Target-P algorithm into account, we propose a mature KEA3 protein with A90 as the most N-terminal AA and a predicted molecular weight of 73.7 kDa.

### Low levels of the deregulated KEA3.3 isoform are sufficient to collapse NPQ in high light

**Figure 2:**
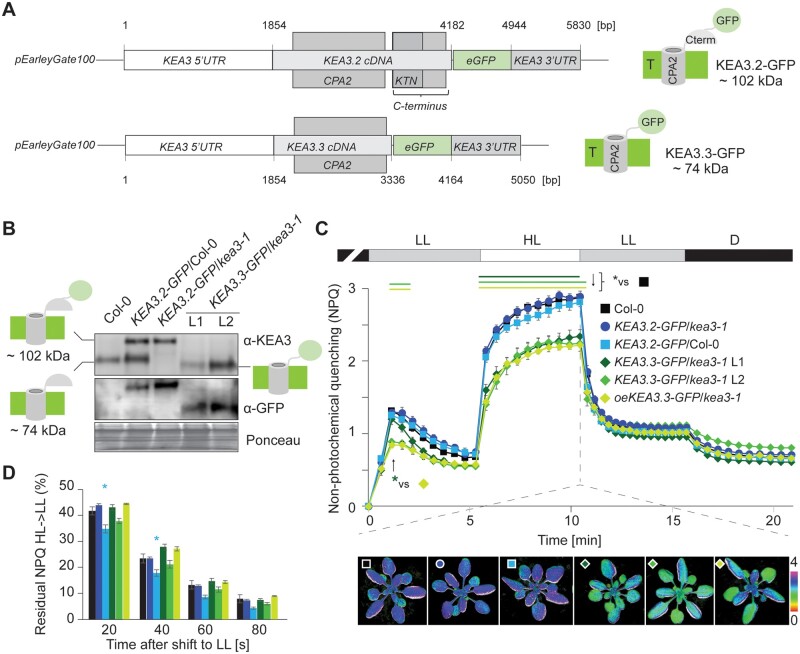
The KEA3 C-terminus is required for full NPQ build-up in high light (HL). A, Scheme of *KEA3.2-GFP* and *KEA3.3-GFP* native expression constructs inserted into *pEarleyGate100* lacking the *35S* promoter. Models right of the schemes show the resulting protein-GFP fusions in the thylakoid membrane (T, thylakoid membrane; CPA2, cation proton antiport 2 domain; KTN, K^+^ transport nucleotide binding domain; Cterm, soluble C-terminus). B, Immunoblots using antibodies against KEA3 (C-terminus) and GFP reveal the presence of the different KEA3 versions in the plant lines. Ponceau Red (Ponceau) stain of membrane prior to immune-detection is shown as a loading control. C, By using the Imaging-PAM, the different lines as described in B and C and *oeKEA3.3*/*kea3-1* L1 as described by Armbruster et al. (2016) were exposed to an alternating light regime of 5-min low light (LL, 90 µmol photons m^−2^ s^−1^), 5-min HL (900 µmol photons m^−2^ s^−1^), 5-min LL and 5-min darkness and NPQ was determined. False color images of NPQ after 5 min in HL are shown below the NPQ traces. Green lines above the NPQ traces indicate where NPQ of *KEA3.3* expressing plants is significantly lower when compared with the WT Col-0 and green asterisk where NPQ of *KEA3.3-GFP/kea3-1* L1 is significantly higher than of *oeKEA3.3*/*kea3-1* as determined by ANOVA and Tukey’s post hoc pairwise comparison with *P* < 0.05. D, NPQ relaxation was determined after the HL to LL shift and is presented as % of fast relaxing NPQ remaining after the indicated times in LL. Asterisks indicate where *KEA3.2-GFP*/Col-0 has faster NPQ relaxation when compared with Col-0 determined by ANOVA and Tukey’s post hoc pairwise comparison with *P* < 0.005. (C + D) Values are averages of *N* = 10 (Col-0, *KEA3.2-GFP*/*kea3-1*), *N* = 8 (*KEA3.2-GFP*/Col-0), and *N* = 5 (*KEA3.3-GFP*/*kea3-*1 L1 and L2). Error bars indicate ±se.

We previously hypothesized that the low NPQ of *oeKEA3.3* plants in high light was the result of proton antiport deregulation in the *oeKEA3.3* plants due to the absence of the regulatory C-terminal domain (Armbruster et al., 2016). However, we did not resolve the potential effects that the high levels of KEA3.3 may have had on NPQ. Thus, we generated plants that expressed either *KEA3.2* or *KEA3.3* as C-terminal GFP fusions from the *KEA3* promoter to obtain plants with WT-like KEA3 protein levels of either GFP-tagged isoform (Figure 2, A). With the newly assigned N-terminus of KEA3 to A90, the KEA3-GFP fusions have predicted molecular weights of 102 (KEA3.2-GFP) and 74 kDa (KEA3.3-GFP, Figure 2, A), respectively. We selected *kea3-1* plants expressing *KEA3.2-GFP* and *KEA3.3-GFP* and Col-0 plants expressing *KEA3.2-GFP* (Figure 2, B and Supplemental Figure S2, A). In line with the phenotype of *oeKEA3.2-GFP* and *oeKEA3.3-GFP* (Armbruster et al., 2016), these plants were visually indistinguishable from WT (Col-0, Supplemental Figure S2, B). For *KEA3.3-GFP* expressed in *kea3-1* (i.e*. KEA3.3-GFP/kea3-1*), we selected two lines which had a similar KEA3 content as WT. While line 1 (L1) accumulated lower KEA3 levels than WT, L2 had slightly elevated levels. Still, KEA3.3 levels in L2 were much lower than in the *oeKEA3.3/kea3-1* line #1 that was previously shown to have nine-fold higher KEA3 content than WT (Supplemental Figure S2, A; Armbruster et al., 2016). The newly generated plants (Figure 2, B and Supplemental Figure S2, B) were measured for NPQ behavior during an alternating light regime of dark to low light to high light, back to low light, and finally dark, together with the WT Col-0 and *oeKEA3.3-GFP/kea3-1* line #1 (Armbruster et al., 2016). The lines that expressed *KEA3.2-GFP* in the WT or *kea3-1* background had very similar NPQ compared with WT. All the three lines expressing *KEA3.3-GFP* had significantly lower NPQ in high light. After transition from dark to low light, NPQ was also lower in the two lines with higher *KEA3.3* levels (i.e. *oeKEA3.3-GFP* and *KEA3.3-GFP* L2) when compared with WT. However, the *KEA3.3-GFP* L1 with lower KEA3.3 levels had a similar transient NPQ as WT and therefore significantly higher NPQ than *oeKEA3.3-GFP* 40 s after transition from dark to low light (Figure 2, C). To characterize the effects of KEA3 isoform and levels on NPQ relaxation, we normalized NPQ to its maximum in high light and minimum in low light (Figure 2, D). This analysis revealed that NPQ relaxation was significantly faster in *KEA3.2-GFP*/Col-0. This line has the highest levels of KEA3.2 of all *KEA3.2* expressing plants (Figure 2, B). Interestingly, it contains more endogenous KEA3 than the WT, suggesting that the additional expression of *KEA3.2-GFP* increases levels of the endogenous KEA3. The acceleration of NPQ relaxation by high KEA3.2 levels was shown previously (Armbruster et al., 2016).

We reasoned that if KEA3.3 was a deregulated version of KEA3, which in contrast to the WT KEA3.2 is not inhibited in high light, NPQ should be likewise decreased by KEA3.3 introduction into WT. Indeed, when we expressed *KEA3.3* from the *KEA3* promoter in Col-0, the resulting plants exhibited reduced NPQ in high light (Supplemental Figure S3, A–D).

**Figure 3:**
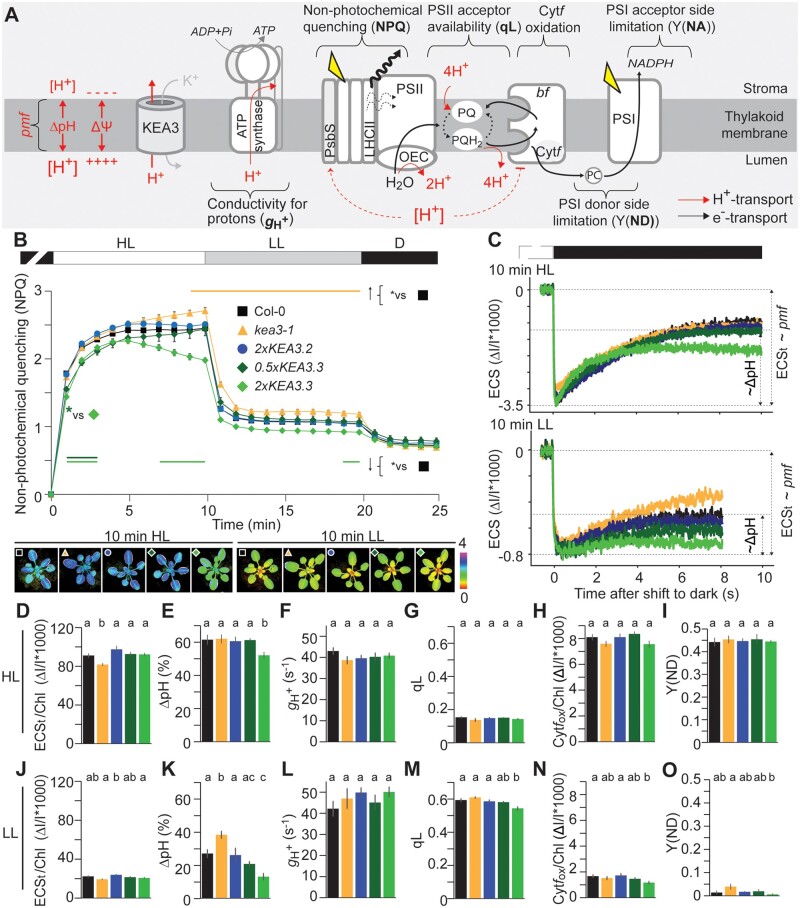
Steady-state photosynthesis responds to KEA3 regulation and protein levels. A, Scheme of the thylakoid membrane depicting the pmf and its components ΔpH and ΔΨ, the feed-back regulatory targets of lumenal protons (PsbS and *bf*, dashed lines) and photosynthetic parameters that were derived in this work. LHCII, light harvesting complex II; PSII, photosystem II, OEC, oxygen evolving complex; PQ, plastoquinone; bf, cytochrome *b_6_f* complex; PC, plastocyanin; PSI, photosystem I. B, NPQ traces of dark-acclimated 5-week-old Col-0 (WT), *kea3-1*, 2x*KEA3.2*, 0.5x*KEA3.3*, and 2x*KEA3.3* during a shift to HL (900 µmol photons m^−2^ s^−1^) for 10 min, subsequent shift to LL (90 µmol photons m^−2^ s^−1^) for 10 min and 5 min dark recovery. Average is shown for *N* = 8 and error bars indicate ±se. The red line above and the green line(s) below the NPQ traces indicate where NPQ of *kea3-1* and the two *KEA3.3* expressing plants, respectively, are significantly different from Col-0 as determined by ANOVA and Tukey’s post hoc pairwise comparison with *P* < 0.05. False color images below the traces show representative plants after 10 min in HL (left panel) and 10 min in LL (right panel). C, Representative ECS traces after 10 min at the given light intensity during the transition from HL (top panel) and LL (bottom panel) to dark. The difference in the ECS signal between light and the transient minimum in dark is ECS_t_, which reflects the amplitude of light-induced pmf. The difference between the minimum in the dark and the recovered ECS signal reflects the fraction of pmf stored as ΔpH. D–O, After reaching steady state the following photosynthetic parameters were determined in HL (D–I) and LL (J–O): ECSt ∼pmf (D, H), the ΔpH fraction of the pmf (E, K), ATP synthase conductivity *g*_H_^+^ (F, L), PSII acceptor availability (G, M), Cyt*f* oxidation state (H, N), and PSI donor site limitation (I, O). D–O, Average is shown for *N* = 8 and error bars indicate ±se. Different letters above bars indicate significant differences between genotypes with *P* < 0.05 as calculated by ANOVA and Tukey’s pairwise multiple comparison.

Together, the data show that the KEA3 C-terminus is needed for the expedited full build-up of NPQ in high light, and strongly support the hypothesis that KEA3.3 constitutes a deregulated proton antiporter. The introduction of KEA3.3 into plants always resulted in decreased NPQ in high light. However, there was a dosage effect of KEA3.3 on the transient NPQ in low light, as higher KEA3.3 levels (i.e. in L2 and *oeKEA3.3*) were required for suppressing the transient NPQ when compared with WT.

### KEA3 activity shapes NPQ activation in high light in a dosage-dependent manner

To continue exploring the role of KEA3 regulation, further work was focused on the lines, in which native KEA3 had been replaced by either KEA3.2-GFP (*KEA3.2-GFP*/*kea3-1*) or KEA3.3-GFP (*KEA3.3-GFP*/*kea3-1* L1 and L2). Protein levels of these lines were determined by gradient Western blots (Supplemental Figure S4, A). This analysis revealed that *KEA3.3-GFP*/*kea3-1* L1 had slightly less than 50% of WT KEA3 levels, while both *KEA3.3-GFP*/*kea3-1* L2 and *KEA3.2-GFP*/*kea3-1* had approximately twice the amount of WT KEA3 (Supplemental Figure S4, A). Consequently, the lines were renamed *0.5xKEA3.3* (*KEA3.3-GFP*/*kea3-1* L1), *2xKEA3.3* (*KEA3.3-GFP*/*kea3-1* L2), and *2xKEA3.2* (*KEA3.2-GFP*/*kea3-1*) for a shortened and more descriptive representation. 77K Chl *a* fluorescence emission spectra of these lines together with WT and *kea3-1* showed no genotype-specific differences in the relative antenna cross sections, and excitation energy distribution between both photosystems (Supplemental Figure S4, B). Likewise, total chlorophyll (Chl) content per leaf area (Supplemental Figure S4, C), the Chl a/b ratio (Supplemental Figure S4, D), and the maximum amplitude of difference transmittance signals of P_700_, the Chl *a* dimer in the PSI reaction center (Supplemental Figure S4, E), and plastocyanin (Supplemental Figure S4, F), both normalized to the Chl content, did not reveal any significant differences. Finally, also the maximum quantum efficiency of PSII in the dark-adapted state (*F*_V_/*F*_M_; Supplemental Figure S4, G) was indistinguishable between WT and all mutant lines.

**Figure 4:**
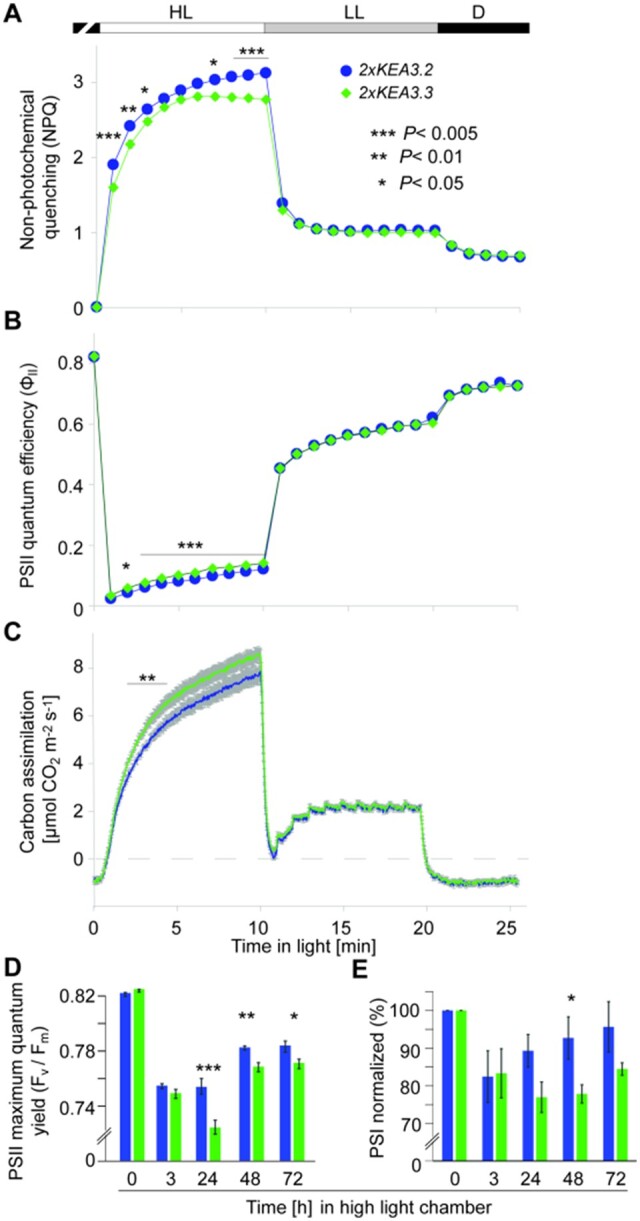
Inhibition of thylakoid proton antiport by the KEA3 C-terminus protects photosystem function in long-term HL. A–C, Chl *a* fluorescence and gas exchange were measured simultaneously of dark-acclimated 5-week-old *2xKEA3.2* and 2x*KEA3.3* during 10-min HL (900 µmol photons m^−2^ s^−1^), 10 min LL (90 µmol photons m^−2^ s^−1^) and 5-min darkness. NPQ (A) and PSII quantum efficiency Φ_II_ (B) were derived and carbon assimilation was calculated per leaf area (C). D–E, Maximum quantum yield of PSII (*F*_v_/*F*_m_, D) and redox-active PSI content (E) of 5-week-old *2xKEA3.2* and *2xKEA3.3* plants were monitored before and at indicated times after shift to a HL phytotron (900 µmol photons m^−2^ s^−1^ with 12 h light/12 h dark photoperiod). E, Functional PSI was determined as P700 (Δ*Ι*/*Ι*) and is represented normalized to the values before the shift. Average is shown for *N* = 6 (A–C) and *N* = 8 (D–E) and error bars indicate ±se. Asterisks indicate significant difference as determined by *t* test with *P < 0.05, **P < 0.01, and **P < 0.005.

We then aimed at a comprehensive characterization of the light-dependent, thylakoid-localized electron and proton transfer reactions (illustrated in Figure 3, A) of these lines during steady-state photosynthesis in high and low light. Initially, we measured Chl *a* fluorescence of dark-acclimated plants during an alternating light regime, which allowed photosynthesis to reach steady state first at high light and subsequently at low light. In line with the results obtained during the shifts from low to high light (Figure 2, C and Supplemental Figure S3, B), NPQ was initially significantly lower in dark-acclimated KEA3.3 containing plants after shift to high light by about 10% when compared with the other lines (Figure 3, B). However, the delay in NPQ induction was slightly, but significantly more pronounced in *2xKEA3.*3 than in *0.5xKEA3.3*. Moreover, also differing from the results of the previous shorter fluctuations, where all *KEA3.3* expressing plants continued to have lower NPQ than the *KEA3.2* expressing lines up to 5 min after shift from low to high light (Figure 2, C and Supplemental Figure S3, B), NPQ levels became very similar between all lines at 5 min after transition from dark to high light (Figure 3, B). The NPQ induction phase was followed by a second phase, in which NPQ continued to increase in *kea3-1* and *0.5xKEA3.3*, remained at the same level in WT and *2xKEA3.2*, but dropped again in *2xKEA3.3* to 80% of WT and *2xKEA3.2*. NPQ levels after 10 min of high light were increased by about 8% in *kea3-1*. The *kea3* mutant persisted to have slightly higher NPQ in the subsequent low light period when compared with WT, while NPQ was decreased in *2xKEA3.3*. Transfer back to darkness collapsed the NPQ differences between lines, which supported that NPQ alterations in the light were due to the pH-dependent NPQ component qE that becomes rapidly switched off in the dark (Quick and Stitt, 1989). The quantum efficiency of PSII was increased in *2xKEA3.3* after 2–3 min in high light and reduced in *0.5xKEA3.3* and *kea3-1* after the transition from high to low light (Supplemental Figure S5, A).

**Figure 5:**
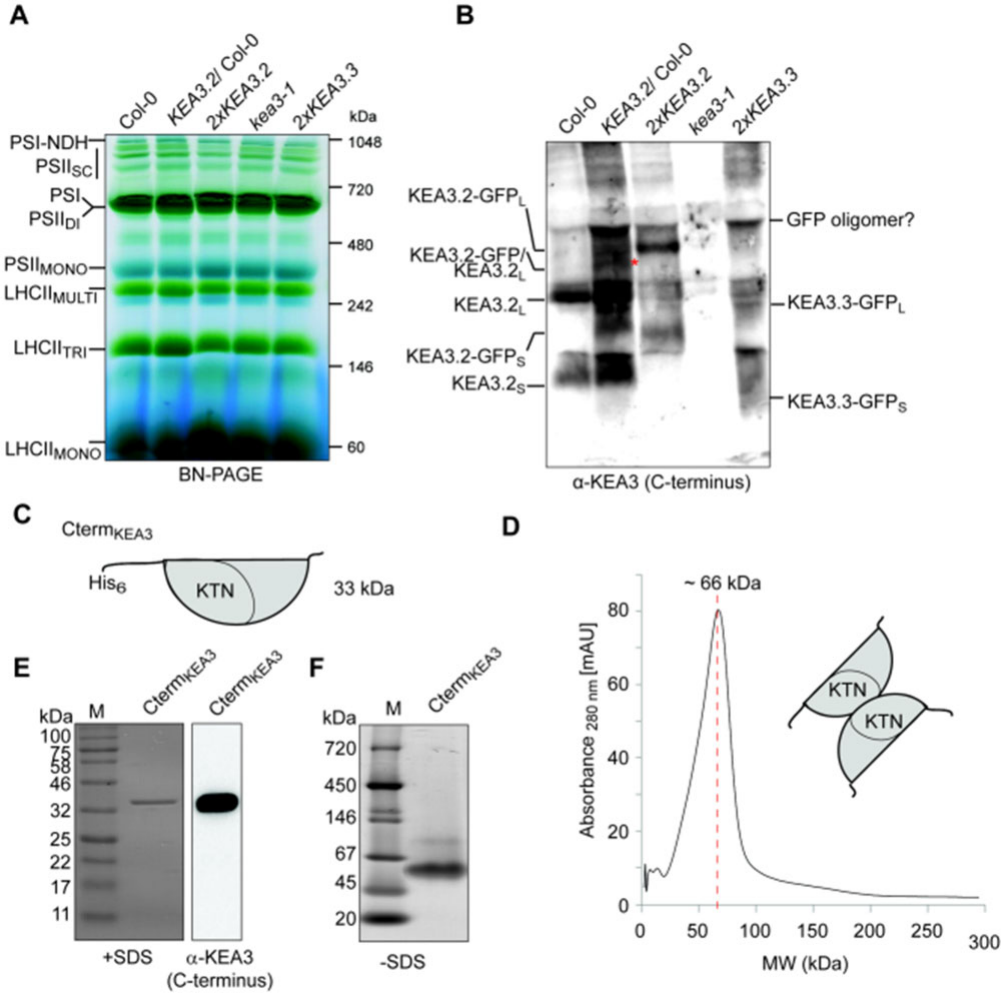
KEA3 homomerizes in the thylakoid membrane via its C-terminus. A, BN PAGE separation of thylakoid complexes from Col-0, *KEA3.2-GFP*/Col-0 (*KEA3.2/*Col-0), *KEA3.2-GFP*/*kea3-1 (2xKEA3.2)*, *KEA3.3-GFP*/*kea3-1* line 2 (*2xKEA3.3*). Labels to the left describe thylakoid complexes migrating at the respective position (LHCII monomer [MONO], trimers [TRI], and multimers [MULTI], PSII monomers [MONO], dimers [DI], and supercomplexes [SC], PSI monomer and PSI-NDH supercomplexes. B, Immunochemical detection of KEA3 using an antibody against the KEA3 C-terminus reveals the presence of smaller and larger KEA3 containing complexes (KEA3_S_ and KEA3_L_, respectively). In *KEA3.2-GFP*/Col-0, which harbors the native KEA3 (KEA3.2) and the GFP-tagged version of KEA3.2, an additional high molecular weight complex can be detected that is in size between the KEA3.2_L_ and KEA3.2-GFP_L_ (red asterisk). *2xKEA3.3* has less KEA3 signals in higher molecular weight complexes. C, Scheme of the recombinant KEA3 C-terminus. D, SEC of the recombinant KEA3 C-terminus shows protein elutes at around 66 kDa, the molecular weight of a dimer. E, The SEC purified KEA3 C-terminus runs slightly above the 32-kDa marker (M) band in the presence of SDS as visualized by Coomassie staining (left panel) and KEA3 antibody (right panel). F, In the absence of SDS, recombinant protein migrates between the 45- and 67-kDa marker bands.

### While *2xKEA3.3* overcompensates a lack of endogenous KEA3, *0.5xKEA3.3* complements the *kea3-1* phenotypes under steady-state conditions

To analyze the size of light-induced pmf and its ΔpH component in steady-state high and low light (900 and 90 µmol photons m^−2^ s^−1^, respectively), we monitored the ECS signal during light to dark transitions (Sacksteder et al., 2000; Cruz et al., 2001). Exemplary non-normalized difference transmittance signals (Δ*I*/*I*) recorded on intact leaves after 10 min of light treatment are shown (Figure 3, C). The ECS signal rapidly decays in the dark and the extent of this decay gives information about the size of the light induced pmf (ECS_t_). The ECS then recovers to a steady-state signal in the dark. The difference between the minimum ECS signal and steady-state ECS signal in the dark corresponds to the fraction of pmf stored as ΔpH (Figure 3, C; Cruz et al., 2001). In high light, ECS_t_, which was normalized to the Chl content of the leaf, was lower in *kea3-1* when compared with the other genotypes, all of which had very similar levels of light induced pmf (Figure 3, D). In line with its decreased NPQ in high light, *2xKEA3.3* had about 10% less of pmf stored as ΔpH when compared with the other genotypes (Figure 3, E). ECS recovery in the dark was slowest in *kea3-1* (*t*_1/2_ of recovery at ∼3.5 s, with WT, 2xKEA3.2 and 0.5xKEA3.3 at ∼2.5 s) and fastest in *2xKEA3.3* (*t*_1/2_ at ∼1.4 s; Supplemental Figure S5, B). The membrane conductivity for protons (*g*_H_^+^) was unchanged between genotypes in high light (Figure 3, F), as were the acceptor availability of PSII expressed as qL, levels of oxidized cytochrome-*f* (Cyt*f*), and PSI donor and acceptor side limitations (Figure 3, G–I and Supplemental Figure S5, C). Together, these results underscore a higher KEA3 activity in *2xKEA3.3* when compared with the other genotypes in high light: Elevated proton export from the lumen via KEA3.3 in exchange for cations decreases the pmf fraction that is stored as ΔpH. This results in a lower proton concentration of the lumen and thus lower NPQ. At high light, these alterations at the level of pmf composition, however, do not affect any of the other measured photosynthetic parameters besides NPQ.

In steady-state low light, ECS_t_ was only slightly, but significantly higher in 2x*KEA3.2* when compared with *kea3-1* and *2xKEA3.3* (Figure 3, J), while the fraction of pmf stored as ΔpH was about 25% increased in *kea3-1* and reduced to about half in *2xKEA3.3* when compared with WT, while the remaining lines were not significantly different (Figure 3, K). Interestingly, ECS recovery in the dark, as expressed by its half-time (*t*_1/2_), was very different between genotypes. All plants expressing KEA3-GFP fusions had a faster recovery of the ECS signal than WT and *kea3-1* (Supplemental Figure S5, D). Similar to high light, *g*_H_^+^ was unchanged between genotypes (Figure 3, L). PSII acceptor availability and Cyt*f* oxidation were both significantly lower in *2xKEA3.3* when compared with WT and 2x*KEA3.2* (Figure 3, M and N). The donor side limitation of PSI was significantly lower in *2xKEA3.3* when compared with *kea3-1* (Figure 3, O), while PSI acceptor side limitation remained unchanged between genotypes (Supplemental Figure S5, E). Collectively, the data show that also in steady-state low light, pmf in *2xKEA3.3* is shifted toward a lower fraction of ΔpH and thus a higher fraction of ΔΨ. This KEA3-induced alteration of pmf composition in low light is accompanied by lower NPQ and slightly, but significantly elevated levels of ETC reduction, as evidenced by a lowered PSII acceptor availability, Cyt*f* oxidation, and PSI acceptor side limitation (Figure 3, M–O).

Together, the data show that in steady-state high and low light, the *kea3-1* photosynthetic phenotypes are complemented by introduction of low levels of KEA3.3 as in *0.5xKEA3.3* and two-fold levels of KEA3.2 as in *2xKEA3.2.* The only exception is the rate of ECS recovery in dark after pre-illumination with low light, which was increased both in *0.5xKEA3.3* and *2xKEA3.2* when compared with Col-0. The fastest recovery was observed for *2xKEA3.3* and the slowest for *kea3-1* (Supplemental Figure S5, B and D), which suggests that deregulation and levels of KEA3 have an additive effect on the ECS recovery in the dark after low light.

To obtain further information about the light dependency of KEA3 regulation, the plants were exposed to increasing light intensities in the form of a light curve and Chl *a* fluorescence and P700 absorption parameters were determined separately. There was no significant difference in the electron transport rate through PSII between genotypes (Supplemental Figure S5, F). *kea3-1* showed increased NPQ and qL in low and medium light when compared with WT (Supplemental Figure S5, G and H). *2xKEA3.3* showed strong differences to WT mostly during high light with decreased NPQ and PSI donor site limitation and enhanced PSI acceptor side limitation at very high, non-physiological light intensities (Supplemental Figure S5, G–I). The data further support that KEA3 activity is restricted under steady-state conditions. Deregulation as in *2xKEA3.3* causes lowered NPQ and results in reduced PSI donor side and increased PSI acceptor side limitation at high light intensities.

### Regulation of KEA3 activity via the C-terminus has photoprotective function but restricts CO_2_ assimilation during photosynthetic induction

To characterize the physiological function of KEA3 regulation via its C-terminus, we continued further analyses with *2xKEA3.3* and *2xKEA3.2*, which have very similar levels of KEA3 and only differ in the presence of the C-terminus. We performed simultaneous Chl *a* fluorescence and gas exchange measurements under controlled ambient CO_2_ levels. Again, *2xKEA3.3* had lower NPQ than *2xKEA3.2* during the initial 3 min in high light and after 7 min (Figure 4, A). NPQ was not different between both lines after transition to low light and in the dark. PSII quantum efficiency was higher in *2xKEA3.3* after 2 min in high light when compared with *2xKEA3.2* and throughout the high light period (Figure 4, B). Intriguingly, we could observe that the lower NPQ and increased PSII quantum efficiency during the induction period of photosynthesis had a positive effect on CO_2_ assimilation, which was significantly increased in *2xKEA3.3* when compared with *2xKEA3.2* between 2 and 4 min after the light was switched on (Figure 4, C). This result demonstrates that the inhibition of KEA3 activity via its C-terminus as in *2xKEA3.2* negatively affects CO_2_ assimilation capacity during photosynthetic induction.

We then hypothesized that KEA3 regulation via its C-terminus may be in place to protect photosystem functions under longer-term high light stress. To test this hypothesis, *2xKEA3.3* and *2xKEA3.2* plants were transferred from growth light to high light and scored for maximum quantum efficiency of PSII (*F*_v_/*F*_m_) and the content of redox-active PSI, as determined from the maximum amplitude of the difference transmittance signal of P_700_. After 3 h in high light, *F*_v_/*F*_m_ and PSI content were clearly decreased when compared with before the shift (Figure 4, D and E). However, there was no significant difference between *2xKEA3.2* and *2xKEA3.3*. After 24 h, both *F*_v_/*F*_m_ and PSI content were further decreased in *2xKEA3.3*, while *F*_v_/*F*_m_ remained the same in *2xKEA3.2* when compared with 3 h after the shift and PSI content even slightly recovered. At 24 h, *F*_v_/*F*_m_ was significantly reduced in *2xKEA3.3* when compared with *2xKEA3.2*. The significant decrease in *F*_v_/*F*_m_ in *2xKEA3.3* continued for 3 d in high light, at which time point the *F*_v_/*F*_m_ values had stabilized in both lines at a lower level than before the high light treatment (Figure 4, D). While PSI content continuously increased again in *2xKEA3.2* throughout the high light treatment, there was little change to PSI content in *2xKEA3.3* (Figure 4, E). At 48 h after the shift, PSI capacity was significantly lower in *2xKEA3.3* when compared with *2xKEA3.2*.

Together, the data show that the regulation of KEA3.2 activity via its C-terminus has a photoprotective role for both, PSII and PSI in high light. However, during photosynthetic induction, plants may benefit from deregulated KEA3 activity, because lower NPQ and an increase of electron transfer reactions boost the capacity for CO_2_ assimilation.

### KEA3 oligomerizes in the thylakoid membrane via its C-terminus

The newly generated *KEA3* lines were employed to obtain information on KEA3 oligomerization in the thylakoid membrane. For this, thylakoid membranes of Col-0, *kea3-1*, *2xKEA3.2*, *KEA3.2*/Col-0, and *2xKEA3.3* were solubilized and separated via Blue Native (BN) PAGE. As expected from the spectroscopic analyses (Supplemental Figure S4, B–G), no significant differences in thylakoid complex composition or stoichiometry were apparent between the different lines (Figure 5, A). When KEA3 was detected on blotted BN-PAGEs, it became apparent that KEA3 is mainly present in one higher molecular weight complex (Figure 5, B). In Col-0, the main KEA3 containing complex (KEA3.2_L_) migrates around the size of the LHCII multimer, which runs slightly above the 242 kDa molecular size marker. The larger complex has about double the molecular weight of the smaller KEA3 containing band (KEA3.2_S_), which migrates close to the 146 kDa marker. In *2xKEA3.2*, which has the KEA3.2 fused to GFP, the main KEA3 containing complex (KEA3.2-GFP_L_) migrates below the PSII dimer at the size marker of 480 kDa. A smaller KEA3 containing band (KEA3.2-GFP_S_) migrates at about half the size of the main KEA3.2-GFP_L_ band. Interestingly, in the *KEA3.2*/Col-0 line, which harbors both the native KEA3.2 as well as the KEA3.2-GFP fusion, an additional high molecular weight band was observed that migrated between KEA3.2_L_ and KEA3.2-GFP_L_ (Figure 5, B, red asterisk). This finding points to the presence of a KEA3.2/KEA3.2-GFP chimeric interaction in *KEA3.2*/Col-0. Together, these analyses of KEA3.2 oligomerization strongly suggest that KEA3.2 dimerizes to form high molecular weight complexes. KEA3.3 in *2xKEA3.3* was also present in a higher molecular weight complex albeit to a much lesser extent than the KEA3.2 versions (Figure 5, B). All lines expressing a GFP-tagged version had an additional high molecular weight complex at the size of PSII dimer/PSI monomer (Figure 5, B).

We then searched for potential protein interaction partners of KEA3 by analyzing the co-immunoprecipitation (Co-IP) results obtained from the *oeKEA3.3-GFP* and *oeKEA3.2-GFP* lines (Supplemental Figure S1 and Supplemental File S1). The strongest candidates for interactions were selected based on their presence in at least 4 (of 7) Co-IP eluates of *oeKEA3.3-GFP* and *oeKEA3.2-GFP* or 2 (of 4) of *oeKEA3.2-GFP* and absence in the Col-0 controls (Supplementary File S1). Those found only in the *oeKEA3.2-GFP* but not in *oeKEA3.3-GFP* eluates were considered as candidates for binding the KEA3.2 C-terminus. Candidates for binding both KEA3 variants were components of the thylakoid FtsH complex, the thylakoid protein kinase STN8 involved in the PSII repair cycle (Bonardi et al., 2005), the geranylgeranyl diphosphate reductase GGR (Tanaka et al., 2010), PsbS, and the γ-subunit of chloroplast ATP synthase AtpC1 (Inohara et al., 1991). Candidate proteins for C-terminus binding were the oxidative stress-related ABC1-like protein 1 (Osa1; Jasinski et al., 2008), the thylakoid membrane insertase Alb3 (Moore et al., 2000), and the thylakoid lumenal protein TLP18.3 involved in PSII repair (Sirpiö et al., 2007). To investigate these interaction candidates further, we repeated the Co-IP experiment, this time with the new lines *2xKEA3.2* and *2xKEA3.3*. The Co-IP results showed that PsbS and FtsH subunits bind to the GFP trap, because they are found in the pellet of Col-0, which does not express a KEA3-GFP fusion, and thus can likely be considered contaminations (Supplemental Figure S6, A). Additionally, gel slices that contained KEA3 high molecular weight complexes were excised from the BN-PAGE (Figure 5, A and B and Supplemental Figure S6, B) and analyzed for their protein composition by MS. The MS analysis of BN-gel slices mainly reproduced the protein blot results, with KEA3 being differentially accumulated depending on size and isoform and lacking from slices of the *kea3-1* mutant (Supplemental Figure S6, B–D). None of the other potential interaction candidates showed a similar pattern as KEA3 (Supplemental Figure S6, E). However, except for Alb3 and TLP18.3, all other proteins were present at much higher levels (more than four-fold) than KEA3 in the analyzed gel slices (Supplemental Figure S6, E). Thus, only Alb3 and TLP18.3 can be entirely excluded as a component of the KEA3 containing high molecular weight complexes by this method. Additionally, we analyzed all proteins that were identified by MS in the two gel slices for a KEA3-dependent distribution, but could not identify any protein with the same accumulation pattern as KEA3 between the different genotypes. This result suggests that there is no thylakoid protein that requires a stoichiometric interaction with KEA3 for the establishment of high molecular weight complexes.

**Figure 6:**
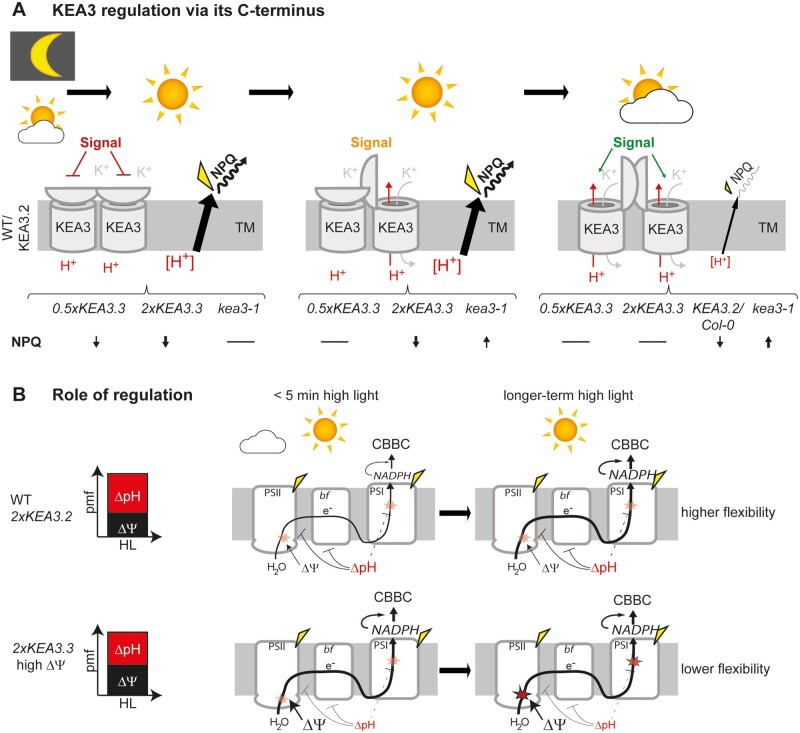
Models of KEA3 regulation and functional implications. A, Scheme depicting the native KEA3.2 dimer in the thylakoid membrane (TM). Dark and LL to HL transitions result in an inhibitory signal (red) that shuts down KEA3.2 activity via a mechanism involving the C-terminal domain. The inhibitory signal ceases after some min in HL (orange) and KEA3.2 becomes partially active. After transition from HL to LL, the signal changes to full activation (green) and KEA3.2 has maximum activity. The model is mainly based on the NPQ results during light fluctuations and the results are summarized below the illustrations as arrows depicting the direction of change when compared with NPQ of Col-0 and *2xKEA3.2* as well as dashes indicating no difference. NPQ serves as indicator for lumenal pH and thus KEA3 activity. HL data are supported by ECS results from steady-state photosynthesis. B, Scheme depicting the functional role of KEA3.2 regulation via its C-terminus. More pmf of *2xKEA3.3* plants is stored as ΔΨ and less as ΔpH when compared with *2xKEA3.2* and WT. In the short term, this has a positive effect on carbon assimilation in HL, potentially because the CBB cycle is activated more rapidly due to higher initial rates of photosynthetic electron transport. High ΔΨ and elevated electron transport rates lead to a gradual increase in charge recombinations and photosystem damage. pmf, light-induced proton motive force; CBBC, Calvin–Benson–Bassham cycle; PSII, photosystem II; *bf*, Cyt *b_6_f* complex; PSI, photosystem I; ΔΨ, membrane potential, ΔpH, proton concentration gradient.

Because KEA3-containing higher molecular weight complexes were much less abundant in *2xKEA3.3*, we hypothesized that the KEA3.2 C-terminus may be responsible for KEA3 dimerization. Thus, we generated recombinant KEA3 C-terminus (Figure 5, C and Supplemental Figure S7), removed residual free Ni^2+^ ions by adding EDTA to avoid interaction of the His_6_-tags and performed size exclusion chromatography (SEC, Figure 5, D). This analysis revealed a peak at ∼66 kDa, which is twice the calculated molecular weight of the recombinant C-terminus. When the main SEC fraction containing the protein was separated in the presence of SDS, the protein was again monomerized and migrated slightly above the 32 kDa size marker band (Figure 5, E). The identity of the band as the KEA3 C-terminus was confirmed by protein blot analysis using the specific KEA3 antibody (Figure 5, E) and MS analysis of the purified protein (Supplementary File S1). The MS analysis revealed that the KEA3-terminus was the main component and contaminating proteins were 100-fold less abundant in the sample (Supplementary File S1). Separation of the KEA3 C-terminus containing SEC fraction via native gel electrophoresis confirmed the migration of the protein as a dimer (Figure 5, F).

Together, the data strongly suggest that KEA3 dimerizes via its C-terminus. In BN-PAGEs, most of KEA3.2 migrates in a higher molecular weight complex, which has approximately double the size of a smaller KEA3.2 containing band (Figure 5, B). These smaller KEA3.2 containing bands may represent monomeric KEA3, migrating at a higher molecular weight than indicated by the protein size marker. The native size marker is composed of soluble, non-membrane proteins, which may exhibit a different migration pattern when compared with the membrane-standing KEA3 with its 13 predicted transmembrane helices (Armbruster et al., 2014). While we obtained some putative interaction partners via MS analysis of KEA3-GFP Co-IPs, the interactions that were further tested could not be confirmed. Currently, we cannot exclude that KEA3 forms other protein–protein interactions besides with itself and may be regulated in this way. Such interactions may be of transient nature. GFP has the tendency to dimerize and oligomerize (Yang et al., 1996; Zacharias et al., 2002) and this behavior may explain the faint KEA3.3-GFP_L_ band present in *2xKEA3.3* and the very high molecular weight complexes just below the PSII dimer present in all the lines expressing GFP-tagged KEA3 versions (GFP-oligomer, Figure 5, B).

## Discussion

### The native KEA3.2 protein dimerizes via its C-terminal domain

The presented data reveal new information about the KEA3 protein. By using multiple experimental approaches in combination with bioinformatic analyses, we located the N-terminus of the mature KEA3 protein in the thylakoid membrane close to the start of the CPA2 transport domain (Figure 1, A–D). In accordance with previous reports, our analyses highlight KEA3.2 as the only detectable isoform in leaves (Figure 1, A; Armbruster et al., 2014, 2016). KEA3.2 forms high molecular weight complexes in the thylakoid membrane that involve the interaction of two KEA3.2 C-terminal domains (Figures 5, B–F, 6, A). We propose that the interaction occurs via the KTN domain, which has been shown to underlie the dimerization of the homologous KefC protein of *Escherichia coli* (Roosild et al., 2009). Full activation of KefC requires interaction of its C-terminus with the auxiliary protein KefF (Miller et al., 2000; Roosild et al., 2009). For KEA3 however, we were not able to identify any stable, stoichiometric interactions with other proteins.

### KEA3.3 represents a deregulated KEA3 version

The steady-state measurements in low and high light demonstrate that *2xKEA3.3* has less pmf stored as ΔpH when compared with WT and *2xKEA3.2*. This finding underscores that KEA3-mediated K^+^-coupled H^+^ antiport out of the lumen is increased in *2xKEA3.3*. Consequently, the presented data strongly support the notion that KEA3.3 represents a deregulated K^+^/H^+^ antiporter. Another variant of KEA3 is the EMS mutagenesis-derived DPGR version of KEA3.2 (Wang et al., 2017). Plants overexpressing *DPGR* showed low pmf and increased *g*_H_^+^ when compared with plants overexpressing *KEA3.2* (Wang and Shikanai, 2019). The Gly422 that is replaced by Arg in DPGR is located in the center of the 11th predicted transmembrane domain of KEA3 and therefore an integral part of the CPA2 domain. The data on DPGR and the site of AA exchange rather suggest alterations at the level of transport specificity/rate of proton slippage. Our data demonstrate that higher KEA3 activity as in *2xKEA3.3* is not accompanied by changes in pmf size and *g*_H_^+^ and therefore support the hypothesis of Wang and Shikanai (2019) that DPGR is not simply an overactive version of KEA3, but likely decouples cation/proton antiport.

### The KEA3.2 C-terminus regulates activity via a light-dependent signal

Intriguingly, the analyses of the different *KEA3.2* and *KEA3.3* expressing lines suggest that KEA3-mediated cation/proton antiport is regulated by a signal that is generated during light transients. We propose that this signal is sensed by the C-terminal domain of the major leaf isoform KEA3.2 and controls antiport activity (Figure 6, A). Our data indicate that KEA3.2 is inactive upon transition from dark or low light to high light (Figure 6, A), then partially active after 5 min in high light, and fully activated after transition from high to low light. This interpretation of KEA3.2 activity is based on the following findings: (1) during photosynthetic induction in high light, NPQ is reduced in plants that express the deregulated KEA3.3 version, and *kea3* mutants do not differ from WT, (2) in steady-state high light, all photosynthetic phenotypes of *kea3* are compensated by the expression of lower levels of *KEA3.3* (in 0.5x*KEA3.3*) and overcompensated in *2xKEA3.3*. The work of Wang and Shikanai (2019) supports our finding that KEA3.2 has some activity in high light. *KEA3.2* overexpressors showed a slight decrease in the ΔpH fraction of the pmf, lower NPQ, and PSI donor side limitation (YND) when compared with WT (Wang and Shikanai, 2019). The hypothesis that *KEA3.2* is strongly activated after a transition to low light is based on the finding (3) that *kea3* mutants have a slow NPQ relaxation (Supplemental Figure S3, B), while NPQ decay is strongly accelerated in plants over-accumulating KEA3.2 as in *KEA3.2/Col-0* in this work (Figure 2, C), and in *KEA3.2* overexpressors (Armbruster et al., 2016). *KEA3.3* plants show no significant alterations of NPQ relaxation when compared with WT or *2xKEA3.2* (Figure 2, D). It has been proposed recently that KEA3 activity directly shapes the kinetics of ECS recovery in the dark (Wang and Shikanai, 2019). Given that this assumption is correct, our data suggest that KEA3.2 is also active directly after transition to dark (Supplemental Figure S5, B–D). One additional interesting observation is that a 5-min illumination at low light prior to the high light exposure (Figure 2, C) leads to a stronger suppression of NPQ in *KEA3.3* expressing plants than when plants are directly shifted from dark to high light (Figure 3, B). Further work will be needed to elucidate whether the differences in NPQ can be explained by alterations at the level of ΔpH between both conditions. However, ECS measurements during photosynthesis transients are very challenging and currently not possible with our set-up.

### Stromal nucleotides as a signal that regulates KEA3 activity through its C-terminus?

The KEA3 C-terminus contains a conserved KTN/RCK domain, which has been shown to bind nucleotides such as ATP, ADP, AMP, and NAD(H) (Schlosser et al., 1993; Roosild et al., 2002; Kröning et al., 2007; Cao et al., 2013) and to gate K^+^ transport processes in response to the binding (Roosild et al., 2002; Cao et al., 2013). For the regulation by stromal nucleotides, the KEA3.2 C-terminus would have to reside in stroma. Previously, results of protease treatments of intact thylakoid membranes have led to conflicting interpretations regarding the KEA3 topology (Armbruster et al., 2016; Wang et al., 2017). Particularly, because protein domains can be protected from proteolysis by other means than their localization in the lumen (as e.g. has been shown for the stromal PSI-D and -E subunits [Zilber and Malkin, 1992; García-Cerdán et al., 2019]), alternative experiments are needed to resolve the localization of the KEA3 C-terminus.

However, if we assume that the KEA3 C-terminus is localized in the stroma and has access to nucleotides, which nucleotides could be regulating KEA3 activity? The stromal production of ATP and NADPH relies directly on light energy. Thus, it is tempting to speculate that these nucleotides and/or their low energy counterparts ADP and NADP^+^ regulate photosynthesis in response to changes in light intensity. Indeed, changes in nucleotide levels were recently linked to alterations in pmf composition (Höhner et al., 2021). Plants lacking the stromal phosphoglycerate dehydrogenase 3 (PGDH3), which have an overreduced NADP(H) and more oxidized NAD(H) pool, show higher ΔΨ at similar pmf when compared with WT.

We have previously proposed that KEA3 antiport is inhibited by a high stromal energy status and is activated in response to energy starvation (Kaiser et al., 2019; Correa Galvis et al., 2020). This hypothesis was based on the following: (1) total leaf ATP and NADP^+^ dynamics measured in response to a sudden decrease in light intensity showed that ATP drops transiently and NADP^+^ increases permanently in low light (Stitt et al., 1989), (2) KEA3 is inactivated in high light and activated after a sudden decrease in light intensity (Armbruster et al., 2016), and (3) KEA3.2 activity appears to be high in plants with low ATP synthase levels and reduced cellular ATP (Correa Galvis et al., 2020). Our data underscore that the main KEA3.2 isoform is inhibited right after a shift from dark to high light (Figures 3, B, 4, A). Considering results from isolated chloroplasts, which showed a rapid increase in NADPH levels within seconds of illumination (Takahama et al., 1981) the hypothesis of KEA3 inactivation by energy replete conditions would still be valid. However, two recent hallmark publications on the use of in vivo sensors for stromal ATP and NADPH measurements in cotyledons during dark-light transitions challenge our view (Voon et al., 2018; Lim et al., 2020). These publications showed that ATP and NADPH levels hardly change within the first minute after transition from dark to light (Lim et al., 2020). While ATP slowly increases over the 3 min in light, NADPH rapidly increases only after a lag of 1 min. In contrast to our previous hypothesis, these in vivo ATP and NADPH data, together with our newly presented data on the regulatory function of the C-terminus, suggest that KEA3 is inactive when ATP and NADPH levels are low and gets activated, once the stroma is charged with increased levels of ATP and NADPH. The finding that KEA3 dimerizes via the C-terminus opens up the possibility of cooperativity of nucleotide binding. Positive cooperativity allows for a strong response at a small change in ligand concentration (Ferrell, 2009). Because all of the recombinant KEA3 C-terminus is found as a dimer (Figure 5, D–F), it is unlikely that dimerization involves nucleotide binding. Also, the C-terminus of the *E. coli* KefC homolog was reported to dimerize independently of nucleotide ligands (Roosild et al., 2009). Altogether, further work is needed to resolve the C-terminus-mediated regulation of KEA3 activity, i.e. regarding the binding of nucleotides to the KEA3 C-terminus; the mechanism of regulation; and the in vivo dynamics of ATP/ADP/AMP, NADP(H), and NAD(H) in response to changes in light intensity.

### De-regulated KEA3.3 does not collapse NPQ

While K^+^/H^+^ antiport activity by KEA3 has not been directly demonstrated, the in planta conversion of ΔpH into ΔΨ by this protein strongly argues for proton cation exchange by KEA3 (Armbruster et al., 2014; Dukic et al., 2019; Wang and Shikanai, 2019; Correa Galvis et al., 2020). K^+^ transport has been proposed based on the homology of KEA3 with known K^+^/H^+^ antiporters (Armbruster et al., 2014) and was supported by K^+^ transport in a K^+^-uptake deficient *E. coli* strain that expressed *KEA3*. K^+^-transport, however, was not accompanied by H^+^ antiport (Tsujii et al., 2019). Due to the indecisive evidence, we cannot exclude that instead of K^+^, KEA3 may be transporting Mg^2+^ or Na^+^ cations, of which Mg^2+^ has been shown to act as proton transport counter-ion during the establishment of ΔpH (Hind et al., 1974). Concentrations of free K^+^, but also of Mg^2+^ or Na^+^ have been reported to be in the millimolar range in the chloroplast (discussed in Cruz et al., 2001; Robinson and Downton, 1984; Ishijima et al., 2003) and thereby in great excess of the chloroplast proton concentration (pH 8–6, 10 nM to 1 µM). Thus, if KEA3.3 was constitutively active with a high turn-over rate, we would expect it to collapse nearly all of the ΔpH established in the light. However, after 10 min in high light ΔpH is reduced by only about 10% when compared with the controls (Figure 3, E). Our current knowledge of chloroplast cation concentrations comes from isolated chloroplasts and may not well reflect in vivo concentrations. These could potentially be lower (Cruz et al., 2001). A genetically encoded K^+^ sensor is now available (Bischof et al., 2017) and can be used in the stroma to quantify K^+^ concentrations and characterize their dynamics in vivo, and potentially reveal, whether KEA3 transports K^+^.

### Role of KEA3 regulation for the protection of photosystems during long-term light stress

The presented results allow us to assign a physiological function to the KEA3 C-terminus, which is the inhibition of KEA3 activity to protect PSII and PSI integrity during long-term light stress (Figures 4, D–E, 6, B). Overactive KEA3, as in *2xKEA3.3*, increases ΔΨ at the expense of ΔpH (Figure 3, E and K). This appears to have consequences for photosynthesis on both levels: Increased ΔΨ favors charge recombinations and thus damage within PSII (Davis et al., 2016), while a low ΔpH fails to appropriately downregulate electron transport from PSII to PSI. High electron transport from PSII that exceeds the demand of the downstream reactions will cause PSI acceptor side limitation. Such limitation has been postulated to result in the damage of the PSI FeS centers by reactive oxygen species (Sonoike, 2011) and can explain the slow PSI recovery in *2xKEA3.3* under prolonged light stress (Figure 4, E). Further evidence for this notion can be found in the light response curves, which reveal lower PSI donor side and increased acceptor side limitation at high light in *2xKEA3.3* when compared with the other lines (Supplemental Figure S5, H–I). The more permanent downregulation of photosynthetic electron transport via photosystem damage in *2xKEA3.3* can be seen as an alternative strategy to synchronize the activities of the light and carbon fixation reactions. However, the downregulation at the level of functional photosystems constrains the flexibility of photosynthesis more strongly than the transient downregulation by the rapidly reversible ΔpH. This holds particularly true for PSI, of which damage is irreversible. While photodamaged PSII undergoes a specialized and highly efficient repair cycle, photoinhibited PSI needs to be fully degraded and replaced by newly assembled complexes (Sonoike, 2011). Recently, it was shown that PSI damage negatively impacts carbon assimilation capacity particularly under light-limited conditions (Lima-Melo et al., 2019). In line with this hypothesis, the function of KEA3.2 regulation through its C-terminal domain would be the preservation of dynamic flexibility within plants photosynthetic responses.

Intriguingly, there is a trade-off to the protective function of the C-terminus. KEA3.2 inhibition during photosynthetic induction limits CO_2_ assimilation capacity (Figure 6, B). We propose that the increased rates of carbon fixation in *2xKEA3.3* when compared with *2xKEA3.2* within the first few minutes of high light (Figure 4, C) stem from higher electron transfer rates, as a result of less pH-dependent feedback downregulation, which in parallel activate the CBB-cycle and the ATP synthase more rapidly (i.e. via the thioredoxin systems, reviewed by Nikkanen and Rintamäki, 2019).

### Conclusion

KEA3 activity uncouples the bioenergetic function of pmf from its feedback downregulatory role. Thus, it is not surprising that KEA3 deregulation leads to photosystem damage under long-term high-light stress. Very intriguing, however is the finding that during photosynthetic induction, KEA3 deregulation boosts carbon assimilation rates. Accordingly, knowledge of the molecular mechanisms that underlie KEA3 regulation may be key for new strategies to enhance plant photosynthesis.

## Material and methods

### Protein analyses

Thylakoids and total leaf proteins were isolated as previously described (Armbruster et al., 2014; Armbruster et al., 2016, respectively). For the separation on SDS-PAGE, a loading buffer with a final concentration of 200 mM Tris (pH 6.8), 8% SDS (w/v), 40% glycerol (v/v), and 200 mM DTT was added to the total leaf sample and diluted 1:1 for the thylakoid samples. Samples were heated at 65°C for 10 min and proteins were separated on SDS-PAGE, blotted onto nitrocellulose, visualized with Ponceau Red (0.1% Ponceau S [w/v] in 5% acetic acid [v/v]) and detected with antibodies specific for the C-terminus of KEA3 (Armbruster et al., 2014). A new antibody was generated against the KEA3 N-terminal AA SAVDVINDLGFDTLT. Additional antibodies that were used were specific for GFP (Chromotek), PsbS (Correa Galvis et al., 2016), FtsH2/FtsH8, and Lhcb3 (Agrisera).

### Transient expression of cTP_KEA3_-YFP fusions in *N. benthamiana* leaves

Coding sequences for the KEA3 cTPs were amplified from the *oeKEA3* constructs (Armbruster et al., 2016) and assembled into pEarleyGate100 (Earley et al., 2006) linearized with *Xba*l and *Xho*l restriction enzymes downstream of the *35S* promoter by using Gibson Assembly (Gibson et al., 2009). Colonies of *Agrobacterium tumefaciens* strain GV3101 transformed with the cTP_KEA3_−YFP constructs were resuspended in induction medium (10 mM MgCl_2_, 10 mM MES-KOH pH 5.6, 150 μM acetosyringone) to an OD_600_ of 0.5. After 2 h at 28°C, suspensions were inoculated onto sections of well-watered *N. benthamiana* leaves by injecting into the bottom side of a punctured leaf (Blatt and Grefen, 2014). Transfected plants were grown for 2 d in room light before detached leaves were analyzed for the localization of the YFP fluorescence signal by confocal microscopy. For microscopy, the Leica TCS SP5 instrument was used with 63×/1.4 objective and water immersion. Fluorophores were excited by using an argon laser at 514 nm with 30% intensity, YFP fluorescence was collected between 524 and 582 nm (930 V gain), and Chl fluorescence between 618 and 800 nm (650 V gain).

### Immunoprecipitation of KEA3-GFP fusions and MS analysis

Thylakoid membranes were solubilized with 1% *n*-dodecyl β-d-maltoside (β-DM) in IP buffer (50 mM HEPES/KOH pH 8.0, 330 mM sorbitol, 150 mM NaCl, 1 mM PMSF, and protease inhibitor cocktail) for 10 min on ice. The supernatant was recovered after centrifugation (10 min, 16,000 *g*, 4°C) and co-incubated with GFP affinity beads (Chromotek) overnight on a wheel at 4°C. The beads were then collected by centrifugation for 2 min at 1,000 *g* and washed six times with IP buffer containing 0.1% β-DM. Proteins were eluted from the matrix by incubation with SDS loading dye buffer. For the identification of precipitated proteins by MS analysis (at the Vincent J. Coates Proteomics/Mass Spectrometry Laboratory at UC Berkeley), eluates were run into SDS-PAGEs and in gel tryptic digestion was carried out. An Agilent 1200 HPLC together with a Thermo-Fisher LTQ XL linear ion trap mass spectrometer was used for a 4-step MudPIT procedure (Washburn et al., 2001). Protein identification and quantification were done with IntegratedProteomics Pipeline (IP2, Integrated Proteomics Applications, Inc., San Diego, CA, USA) using ProLuCID/Sequest, DTASelect2, and Census (Tabb et al., 2002; Xu et al., 2006; Cociorva et al., 2007; Park et al., 2008). Tandem mass spectra were extracted into ms1 and ms2 files from raw files using RawExtractor (McDonald et al., 2004) and were searched against the Arabidopsis (*A. thaliana*) protein database supplemented with sequences of common contaminants (Peng et al., 2003). The Arabidopsis database was downloaded from arabidopsis.org. An in silico digest of the full length KEA3.2 protein sequence was performed by using the Expasy Peptide mass server (https://web.expasy.org/peptide_mass/) with default settings.

### Plant material, propagation, and growth conditions

The *kea3-1* (Gabi_170G09) T-DNA insertion line and the overexpression lines *oeKEA3.2* and *oeKEA3.3* in *kea3-1* have been described previously (Armbruster et al., 2014, 2016). Plants expressing either *KEA3.2-GFP* or *KEA3.3-GFP* from the *KEA3* promoter were generated as follows: *KEA3.2-GFP* and *KEA3.3-GFP* coding sequences were amplified from the *oeKEA3* constructs (Armbruster et al., 2016) by using the primer sequences described in Supplemental Table S1, and then assembled downstream of the *KEA3 5′UTR* (1,854 bp) and upstream of the *KEA3 3′ UTR* (104 bp) into the *pEarlyGate*100 vector (Earley et al., 2006) cut with the two restriction enzymes *BamH*I and *Xba*I by using Gibson assembly (Gibson et al., 2009). *BamH*I cuts upstream of the 35S promotor, which leads to its removal from the final construct. WT Arabidopsis (*A. thaliana*) accession Col-0 and *kea3-1* plants were transformed with the resulting *KEA3.2-GFP* and *KEA3.3-GFP* constructs by *A. tumefaciens*-mediated floral dip (Clough and Bent, 1998). Individual T1 transgenic plants were selected based on their resistance to Basta on soil or 0.8% sterile agar media containing 1× MS nutrient solution without additional sucrose.

Col-0 and transgenic seeds were sown on soil, and grown in long day (LD, 16 h light/8 h darkness at 20°C/6°C, 60%/75% relative humidity, respectively) at 250 µmol photons m^−2^ s^−1^ for 7 d. Then plants were moved to a growth light of 120–150 µmol photons m^−2^ s^−1^ and 16°C night temperature. After 2 weeks, plants were pricked into individual pots. All measurements were performed with plants that were 4–5 weeks old. Long-term high and fluctuating light treatments were performed in a climate chamber set to 900 µmol photons m^−2^ s^−1^ (HL) or fluctuations of 4 min 90 and 1 min 900 µmol photons m^−2^ s^−1^ (FL; photoperiod of 12 h light/12 h dark; no change of climate settings).

### Chl *a* fluorescence and P700 measurements

For Chl a fluorescence analyses during light fluctuations, the Imaging PAM (Walz GmbH, Effeltrich, Germany) was used. Saturation light pulses (setting: 10) were applied after dark acclimation (for Fm) and illumination (for Fm′). The NPQ at a given time point during the light treatments was calculated as (Fm − Fm′)/Fm′ and ΦII as (Fm′ − Fs)/Fm′.

For steady-state measurements of Chl *a* fluorescence and light response curves, the fiberoptics version of the Dual-PAM-100 (Walz GmbH) was used. Plants were dark-acclimated for 30 min prior to the measurement. Then, under light-limited conditions, the light intensity was increased in 150 s intervals. Under light-saturated conditions above 500 µE m^−2^ s^−1^, the light intensity was increased every 60 s. Linear electron transport rates (ETRII) were derived from the quantum yield Y(II) of PSII according to Genty et al. (1989). The fraction of open PSII centers, qL, was calculated according to Kramer et al. (2004).

PSI measurements were performed with the plastocyanin-P_700_ version of the Dual-PAM instrument (Schöttler et al., 2007), which allows the deconvolution of absorbance changes arising from plastocyanin and PSI. Plants were directly taken from the controlled environment chambers and measured without dark adaptation, to avoid an acceptor-side limitation when the maximum content of redox-active PSI was determined. As an in vivo measure for the contents of plastocyanin and PSI, the maximum amplitudes of the difference transmittance signals (Δ*I*/*I*) of P_700_ and plastocyanin were used. After 8 s of pre-illumination with far-red light, to selectively excite PSI and oxidize P_700_ and plastocyanin, a short saturating pulse of actinic light was applied, and the maximum amplitude at the onset of the saturating pulse was quantified. For light response curves of PSI parameters, the light intensity was slowly increased as described above for the Chl *a* fluorescence measurements. The fraction of PSI reaction centers limited at the donor side, Y(ND), or the acceptor side, Y(NA), was determined according to Schreiber and Klughammer (2016).

77K Chl *a* fluorescence emission spectra were measured using a F-6500 fluorometer with a red-sensitive photomultiplier (Jasco Inc., Groß-Umstadt, Germany). Leaf discs were rapidly frozen and ground in liquid nitrogen, and immediately after thawing diluted in a buffer (330 mM sorbitol, 30 mM KCl, 5 mM MgCl_2_, 30 mM HEPES pH 7.6) to a Chl concentration of less than 5 µg chlorophyll ml^−1^, to avoid re-absorbance of Chl *a* fluorescence emitted from PSII by PSI. The sample was excited at 430 nm wavelength with a bandwidth of 10 nm, and the emission spectrum was recorded between 655 and 800 nm in 0.5 nm intervals with a bandwidth of 1 nm. All signals were normalized to the emission maximum of PSII-LHCII at 686.5 nm wavelength.

### Electrochromic shift and Cyt*f* oxidation state measurements

The thylakoid membrane conductivity for protons (gH^+^) was used as a measure for ATP synthase activity. It was determined on intact leaves from the decay kinetics of the electrochromic shift (ECS) signal during a short interval of darkness. The leaves were pre-illuminated for 10 min with saturating light (900 μmol photons m^−2^ s^−1^), so that photosynthetic electron transport and ATP synthase activity were not limited by ATP and NADPH consumption by the CBB cycle. The saturating illumination was interrupted by 15-s intervals of darkness, and the rapid first phase of the decay kinetic of the ECS during the first 200 ms of darkness was fitted with a single exponential decay function. The reciprocal value of the time constant was used as a measure of ATP synthase activity. The maximum amplitude of the ECS during the first phase of its relaxation kinetic was also used as a measure for the total light-induced pmf across the thylakoid membrane (ECS_t_), and pmf partitioning into ΔpH and ΔΨ was resolved by analyzing the slowly relaxing phase of the ECS between 1 and 15 s of darkness as described (Takizawa et al., 2007). Between five and nine repetitive measurements of the dark-interval relaxation kinetics were averaged to increase the signal-to-noise ratio. After completing the measurements at 900 µmol photons m^−2^ s^−1^, the light intensity was decreased to 88 µmol photons m^−2^ s^−1^, and plants were given another 10 min to adapt to the new light conditions, before repetitive measurements were started again. Due to the smaller signal-to-noise ratio at the lower light intensity, at least 16 dark interval relaxation kinetics were averaged. The half time (*t*_1/2_) of the ECS recovery in the dark was determined by performing an exponential fit starting at the ECS minimum in the dark throughout the recovery phase by using Python and the curve_fit function of SciPy.

The redox state of cytochrome *f* (Cyt*f*) was determined in parallel to the ECS measurements. Here, the amplitude of the difference transmittance signal between the oxidized state in saturating light (900 photons m^−2^ s^−1^) or low light (88 µmol photons m^−2^ s^−1^) and the fully reduced state reached within 500 ms in darkness was used as a measure of total redox-active Cyt*f*. Finally, to take differences in leaf Chl content into account, the amplitudes of ECS_t_ and the Cyt*f* difference transmittance signal were normalized to the Chl content per leaf area (Rott et al., 2011). All signals were simultaneously measured between 505 and 570 nm wavelength using the KLAS-100 spectrophotometer (Walz GmbH) and deconvoluted as previously described (Rott et al., 2011).

### Gas exchange measurements

Gas exchange was measured with the LI-COR 6400 gas exchange system coupled to the Imaging PAM (Walz GmbH) that served as a light source and simultaneously recorded Chl *a* fluorescence. Plants were dark-acclimated for 30 min, inserted into the Whole Arabidopsis Chamber, and exposed to 10 min of 900 μmol m^−2^ s^−1^, 10 min of 90 μmol m^−2^ s^−1^, and 5 min of darkness. The temperature was set to 20°C and humidity to 60%. The flow was set to 500 μmol s^−1^ and CO_2_ concentration to 400 μmol mol^−1^. The CO_2_ assimilation data were normalized on rosette size, calculated from Imaging PAM-images of Chl a fluorescence via ImageJ. To record Fm and Fm′, saturation pulses (setting: intensity, 10, length, 480 ms) were applied every 60 s throughout the measurement. Significant differences were determined via one-way ANOVA and subsequent Tukey’s post hoc multiple comparison.

### BN-PAGE and LC–MS/MS-based quantitative proteome analyses of KEA3-containing gel slices

For BN polyacrylamide gel electrophoresis, thylakoid membranes were solubilized with 0.7% β-DM (w/v) and separated by BN gels (Invitrogen) according to Peng et al. (2003). A native protein marker (Serva) was separated for size comparison. BN-slices were excised and digested as described before (Morgan et al., 2008). Further sample processing and LC–MS/MS data acquisition were performed as described previously (Lassowskat et al., 2017). LC–MS/MS analysis was performed by using an EASY-nLC 1200 (Thermo Fisher) coupled to a Q Exactive HF mass spectrometer (Thermo Fisher). Separation of peptides was performed on 17 cm frit-less silica emitters (New Objective, 0.75 µm inner diameter), packed in-house with reversed-phase ReproSil-Pur C_18_ AQ 1.9 µm resin (Dr. Maisch). The column was constantly kept at 50°C. Peptides were eluted in 115 min applying a segmented linear gradient of 0%–98% solvent B (solvent A 0% ACN, 0.1% FA; solvent B 80% ACN, 0.1% FA) Please provide the expansions for CAN, FWHM, and IPTG. at a flow rate of 300 nL/min. Mass spectra were acquired in data-dependent acquisition mode according to a TOP15 method. MS spectra were collected by the Orbitrap analyzer with a mass range of 300–1759 *m/z* at a resolution of 60,000 FWHM, maximum IT of 55 ms, and a target value of 3 × 10^6^ ions. Precursors were selected with an isolation window of 1.3 *m/z* and HCD fragmentation was performed at a normalized collision energy of 25. MS/MS spectra were acquired with a target value of 10^5^ ions at a resolution of 15,000 FWHM, maximum injection time of 55 ms, and a fixed first mass of *m/z* 100. Peptides with a charge of +1, >6, or with unassigned charge state were excluded from fragmentation for MS^2^, dynamic exclusion for 30 s prevented repeated selection of precursors.

Processing of raw data was performed using the MaxQuant software version 1.6.17.0 (Cox and Mann, 2008). MS/MS spectra were assigned to the Araport11 protein database. During the search, sequences of 248 common contaminant proteins as well as decoy sequences were automatically added. Trypsin specificity was required and a maximum of two missed cleavages was allowed. Carbamidomethylation of cysteine residues was set as fixed, oxidation of methionine, deamidation, and protein N-terminal acetylation as variable modifications. A false discovery rate of 1% for peptide spectrum matches and proteins was applied. Match between runs was enabled.

### Heterologous expression and purification of the soluble KEA3 C-terminus

The construct used for the expression of the KEA3 C-terminus in *E. coli* was described previously (Correa Galvis et al., 2020). Rosetta cells transformed with the construct were grown at 37°C overnight. The cultures were diluted to obtain OD_600_ of 0.8 and protein expression was induced by the addition of 1 mM IPTG. After 3.5 h, cells were harvested by centrifugation, resuspended in the lysis buffer (50 mM HEPES pH 8, 300 mM NaCl, 5 mM imidazole, 10% glycerol), and disrupted by sonication on ice. Soluble proteins were obtained by centrifugation at 15,000 *g* and subsequent filtering through a filter of 0.2 µM pore sizes. C-term_KEA3_ was purified by Ni-NTA agarose (Qiagen) and elution with 300 mM imidazole in the purification buffer, upon washing the Ni-NTA agarose with wash buffer containing 30 mM imidazole. The eluate was subsequently treated with 2 mM EDTA to remove residual Ni^2+^ ions. Next, SEC was performed by using the Superdex 200 Increase 10/300 GL column (GE Healthcare), which was calibrated with low and high molecular weight calibration kits according to manufacturer’s instructions. After SEC, protein from the fractions containing C-term_KEA3_ were concentrated and the buffer was exchanged to phosphate-buffered saline at pH 8 using Amicon Ultra Centrifugal filters with a 10-kDa cutoff (FisherScientific). Purified proteins were then separated by native or SDS-PAGE and analyzed by MS.

For MS analysis, proteins were reduced with DTT, modified with iodoacetamide, and digested on column with trypsin (Promega) in 100 mM ammonium bicarbonate for 14 h at 37°C. Peptides were eluted with 0.5 M NaCl. Trifluoroacetic acid (10%) was used for peptide acidification to pH < 3.0. The peptide mixture was purified and desalted on C18 SEP-Pak columns (Tecknokroma). Measurements were performed on a Q Exactive *Plus* mass spectrometer coupled with a nLC1000 nano‐HPLC (both Thermo Scientific). Quantitative analysis of MS/MS measurements was performed with the MaxQuant software (Cox and Mann, 2008) and the Mascot search engine was used to annotate peptide sequences using Arabidopsis TAIR10 genome annotation database.

### Data availability

MS raw data of BN-PAGE slices were deposited via JPOST (Moriya et al., 2019) under the following identifiers JPST000877 and PXD019865. The data are available under the following link for reviewers and will be made publicly available upon publication. URL: https://repository.jpostdb.org/preview/313472735fa8eb9d93ec1, access key: 6473.

## Accession numbers

Sequence data from this article can be found in the GenBank/EMBL data libraries under accession numbers KEA3 (AT4G04850), PSBS (AT1G44575), FTSH2 (AT2G30950), FTSH8 (AT2G30950), GGR (AT1G74470), ATPC1 (AT4G04640), OSA1 (AT5G64940), ALB3 (AT2G28800), TLP18.3 (AT1G54780), LHCB3 (AT5G54270).

## Supplemental data

The following materials are available in the online version of this article.

**Supplemental Figure S1.** Immunoprecipitation (IP) with a GFP trap removes GFP-tagged KEA3 quantitatively from solubilized thylakoids.

**Supplemental Figure S2.** Selection of *KEA3.2* and *KEA3.3* native expression lines.

**Supplemental Figure S3.** Low levels of KEA3.3 in the WT background decrease NPQ in high light.

**Supplemental Figure S4.** Characterization of *kea3-1* lines expressing *KEA3.2-GFP* or *KEA3.3-GFP.*

**Supplemental Figure S5.** *2xKEA3.3* overcompensates the photosynthetic phenotypes of *kea3-1* particularly at high light intensities.

**Supplemental Figure S6.** No clear protein interaction partner can be assigned for KEA3.

**Supplemental Figure S7.** Purification of recombinant KEA3 C-terminus from *E. coli*.

**Supplemental Table S1.** Primer names, purpose, and DNA sequences used for the generation of native expression lines

**Supplemental Data set S1.** Proteomics data of Co-IP, BN slices, and recombinant KEA3 C-terminus.

## Supplementary Material

kiab135_Supplementary_Data

## Notes

V.C.G. generated the transgenic plants. X.C., E.T., J.R., S.M., and M.U. performed the western blot analyses. U.A. and S.M. performed the co-immunoprecipitations. M.U. generated the cTP-YFP fusions and localized fusions using confocal microscopy. S.M. performed the BN-PAGE analysis. J.E. and I.F. performed the MS analysis on BN slices. M.U. produced the recombinant protein in *Escherichia coli* and performed size exclusion chromatography together with M.L. and A.S. E.S. and A.S. performed the MS analysis of recombinant protein. S.M., M.U., and E.T. performed the Chl *a* fluorescence measurements during light fluctuations. M.A.S. measured the 77K Chl *a* fluorescence emission spectra, ECS kinetics, and Cyt*f* redox state. M.A.S. and U.A. performed the Chl *a* fluorescence and P700 light response curves and steady-state measurements, respectively. T.v.B. carried out simultaneous CO_2_ assimilation and Chl *a* fluorescence measurements. U.A. wrote manuscript with help from all authors.

The author responsible for distribution of materials integral to the findings presented in this article in accordance with the policy described in the Instructions for Author (https://academic.oup.com/plphys/pages/general-instructions) is: Ute Armbruster (armbruster@mpimp-golm.mpg.de).
