# Erratum to: Functional characterization of proton antiport regulation in the thylakoid membrane

**DOI:** 10.1093/plphys/kiab582

**Published:** 2021-12-27

**Authors:** Michał Uflewski, Sarah Mielke, Viviana Correa Galvis, Thekla von Bismarck, Xiaoheng Chen, Enrico Tietz, Jeremy Ruß, Marcin Luzarowski, Ewelina Sokolowska, Aleksandra Skirycz, Jürgen Eirich, Iris Finkemeier, Mark Aurel Schöttler, Ute Armbruster

*Plant Physiology*, kiab135, https://doi.org/10.1093/plphys/kiab135

Due to a production error, a typo was introduced in the title of this article after the authors reviewed the proof. The title “Functional characterization of protonantiport regulation in the thylakoid membrane” has been corrected to “Functional characterization of proton antiport regulation in the thylakoid membrane.” We apologize to the authors for the error.

