# Supplementary material for: Functional characterization of proton antiport regulation in the thylakoid membrane
Source: Plant Physiol. 2021 Mar 20;187(4):2209–29. doi: 10.1093/plphys/kiab135 (PMC8644300; doi:10.1093/plphys/kiab135)
Supplement: kiab135_Supplementary_Data [file kiab135_supplementary_data.zip › pp.01599.2020-s01.pdf]

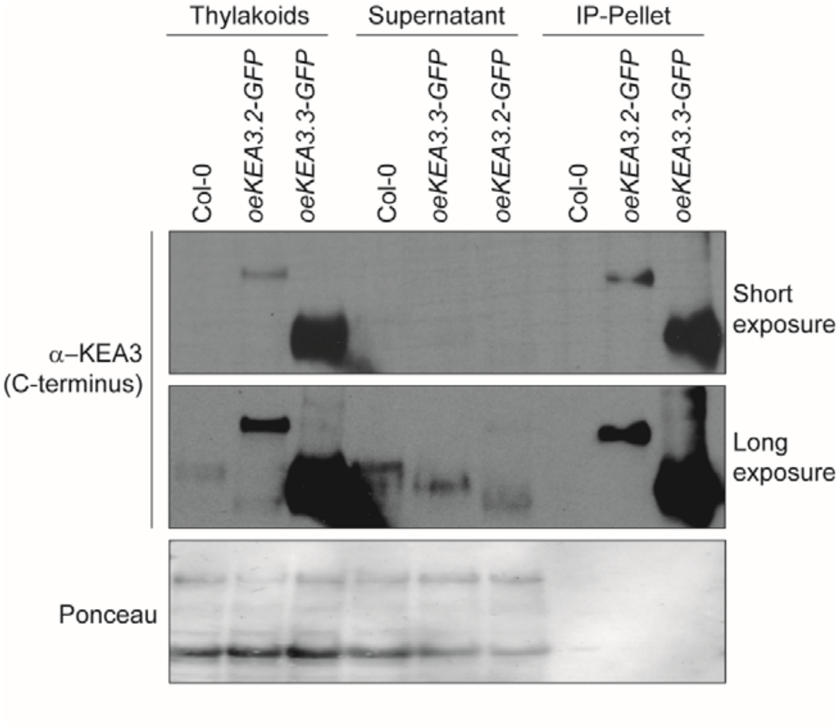

Supplemental Figure S1. Immunoprecipitation (IP) with a GFP trap removes GFP-tagged KEA3 quantitatively from solubilized thylakoids.

Proteins from thylakoids of Col-0, *oeKEA3.2-GFP/kea3-1* and oe*KEA3.3-GFP/kea3-1* (Armbruster et al., 2016), supernatant, and washed IP pellet after solubilization with ß-DM and incubation with a GFP trap, were separated by SDS-PAGE and immuno-detected with the specific C-terminal KEA3 antibody. Ponceau Red (Ponceau) stain of membrane prior to immune-detection is shown as a loading control. Proteins from the IP pellet were subjected to tryptic digest and analyzed via LC MS/MS.

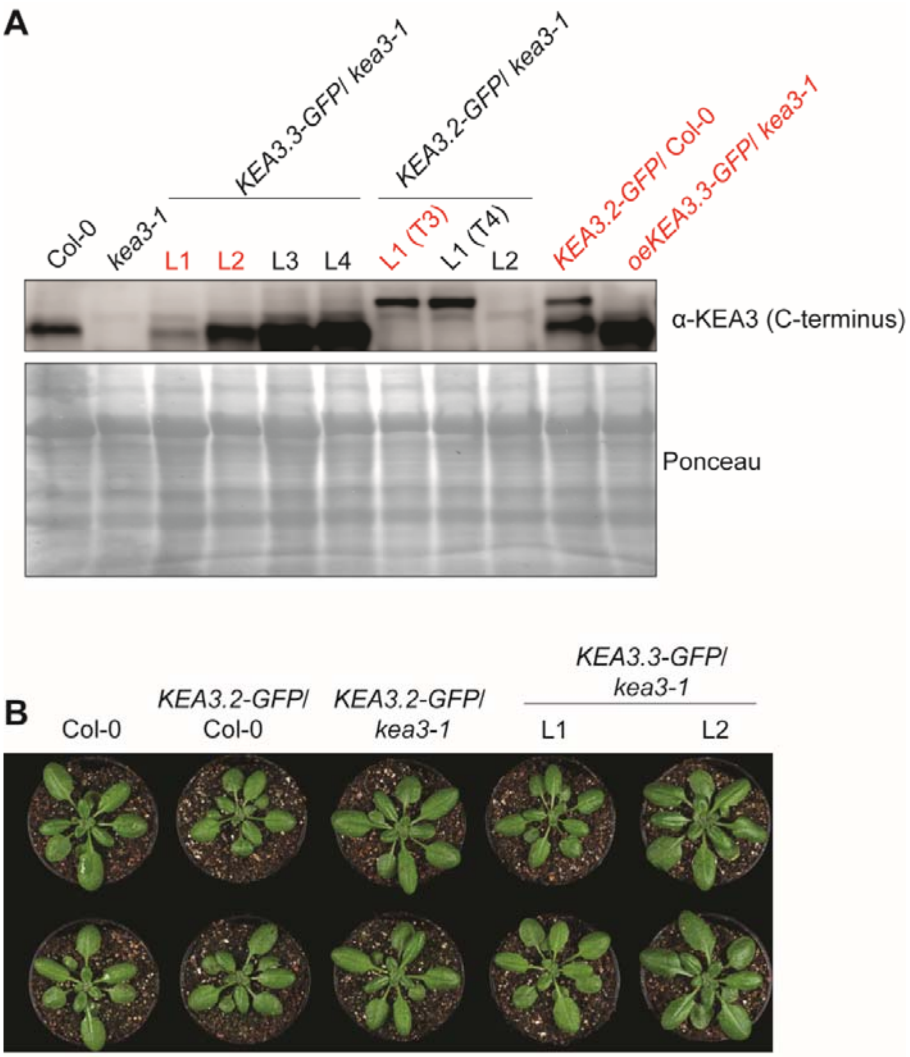

Supplemental Figure S2. Selection of *KEA3.2* and *KEA3.3* native expression lines.

(A) Homozygous T3 lines were analyzed for their protein content by immunochemical detection using the C-terminal KEA3 antibody. Lines that were selected for further analysis are marked in red. Ponceau staining of membrane prior to immunodetection is shown as loading control. (B) Picture of five-week-old Col-0, *KEA3.2-GFP*/Col-0, *KEA3.2-GFP/kea3-1*; *KEA3.3-GFP/kea3-1* L1 and L2.

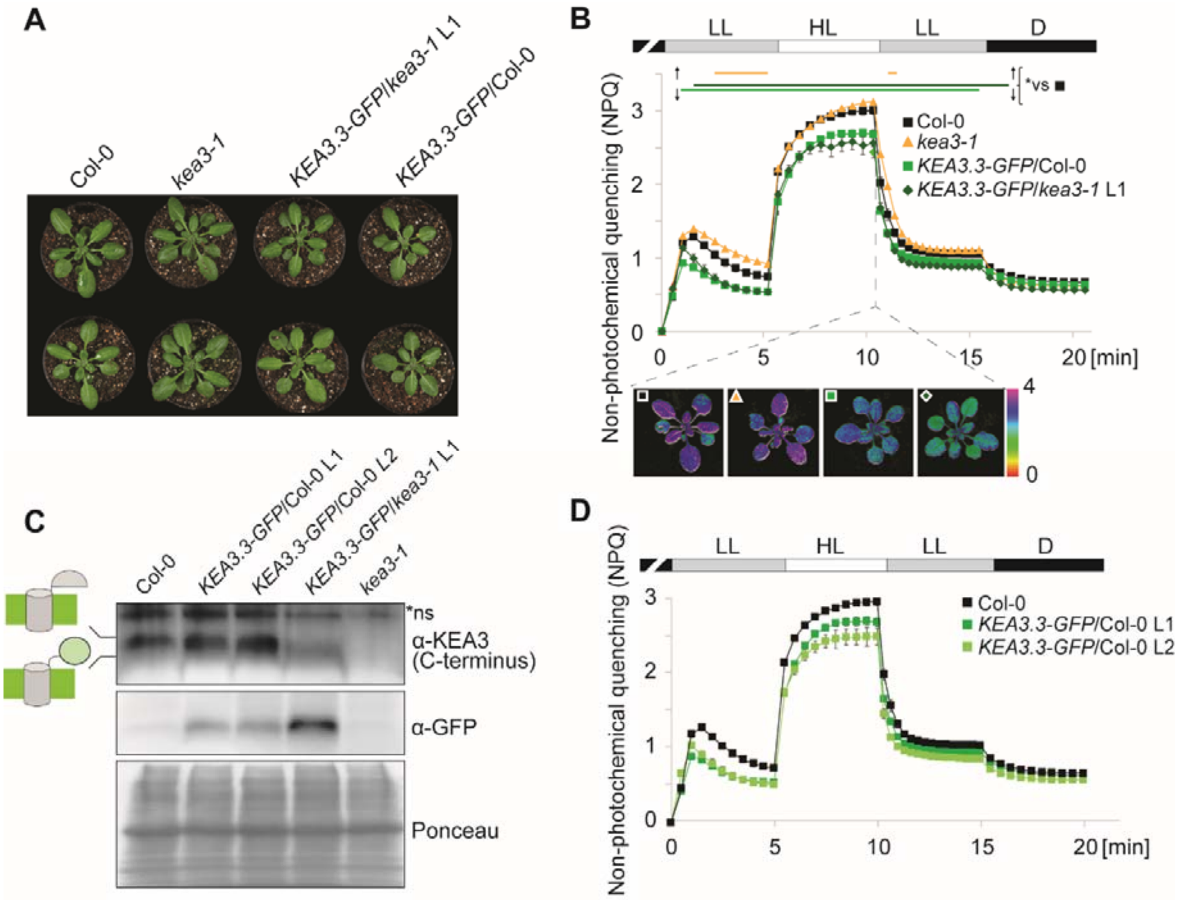

Supplemental Figure S3. Low levels of *KEA3.3* in the WT background decrease NPQ in high light

(A) Pictures of five-week-old WT (Col-0), *kea3-1, KEA3.3-GFP*/*kea3-1* line 1 (L1) and *KEA3.3-GFP*/Col-0. (B) By using the Imaging-PAM, the different lines as described in A were exposed to an alternating light regime of 5 min low light (LL, 90 µmol photons m^{-2} s^{-1}), 5 min high light (HL, 900 µmol photons m^{-2} s^{-1}), 5 min LL and 5 min darkness. Chl *a* fluorescence was measured to determine non-photochemical quenching (NPQ). False color images of NPQ determined after 5 min in HL are shown below the NPQ traces. Values are averages of N=10 (Col-0), N=12 (*KEA3.3-GFP*/Col-0) and N=6 (*kea3*-1, *KEA3.3-GFP*/ *kea3-1* L1). Error bars indicate SE. Green lines above the NPQ traces indicate where NPQ of *KEA3.3* expressing plants is significantly lower and the red line where *kea3-1* NPQ is significantly higher as compared to Col-0 as determined by ANOVA and Tukey posthoc pairwise comparison with *$P< 0.05$. (C) Immunoblots using antibodies against KEA3 (C-terminus) and GFP antibodies reveal the presence of the two KEA3 versions in the different plants. Ponceau Red (Ponceau) stain of membrane prior to immune-detection is shown as a loading control. (D) Two independent lines of *KEA3.3-GFP* in Col-0 were analyzed together with Col-0 and showed similarly reduced NPQ in LL and in HL. Values are averages of N=10 (Col-0), N=12 (*KEA3.3-GFP*/Col-0 L1) and N=3 (*KEA3.3-GFP*/Col-0 L2). Error bars indicate SE.

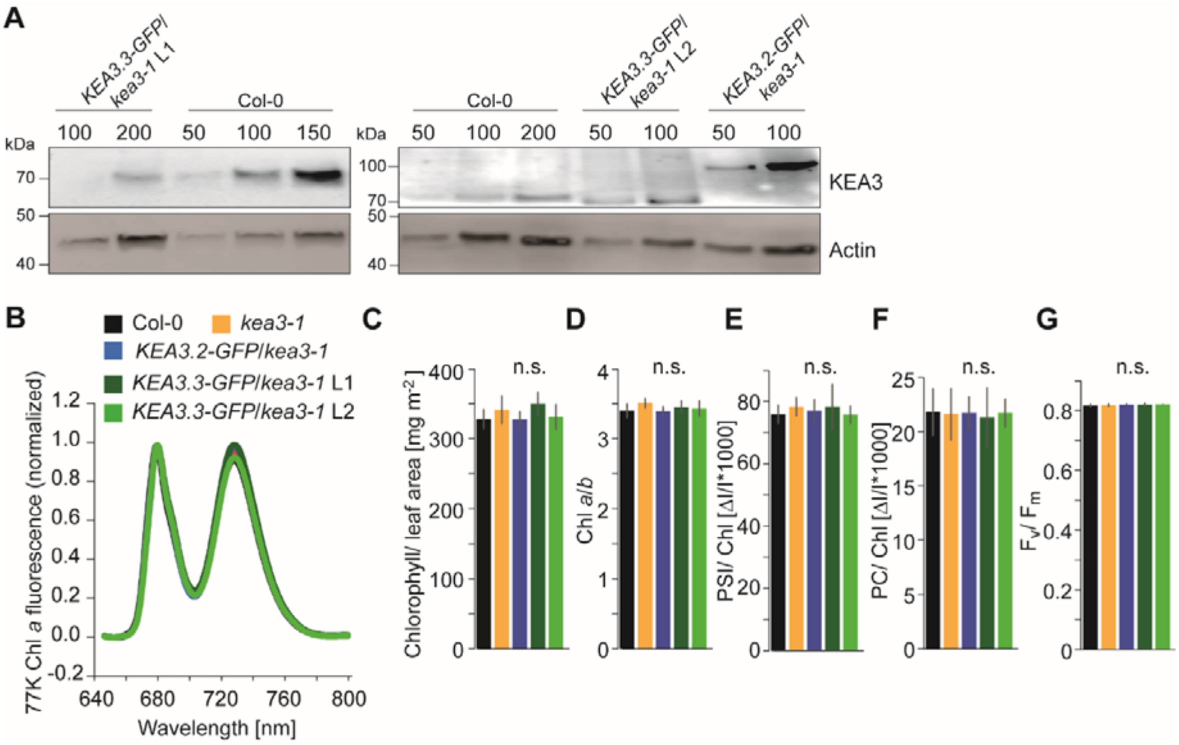

Supplemental Fig. 4

Supplemental Figure S4. Characterization of *kea3-1* lines expressing *KEA3.2-GFP* or *KEA3.3-GFP.*

(A) Gradient Western blots were performed to determine protein levels in *KEA3.3-GFP/kea3-1* L1 and L2 and *KEA3.2-GFP/kea3-1*, revealing that *KEA3.3-GFP/ kea3-1* L1 carries about half of the native KEA3 of WT (Col-0), while the other two lines have about twice the native levels. (B) 77K Chl *a* fluorescence emission measurements show no strong differences between genotypes, neither do chlorophyll content per leaf area, Chl *a*/*b* ratio (D), PSI (E) and plastocyanin (F) content and PSII maximum quantum efficiency (F_v/F_m, G). (B-G) Average is shown for N = 6. (C-G) Error bars indicate ± SD. ANOVA and Tukey posthoc pairwise comparison revealed no statistical differences between the genotypes with $P < 0.05$ (not significant, n.s.).

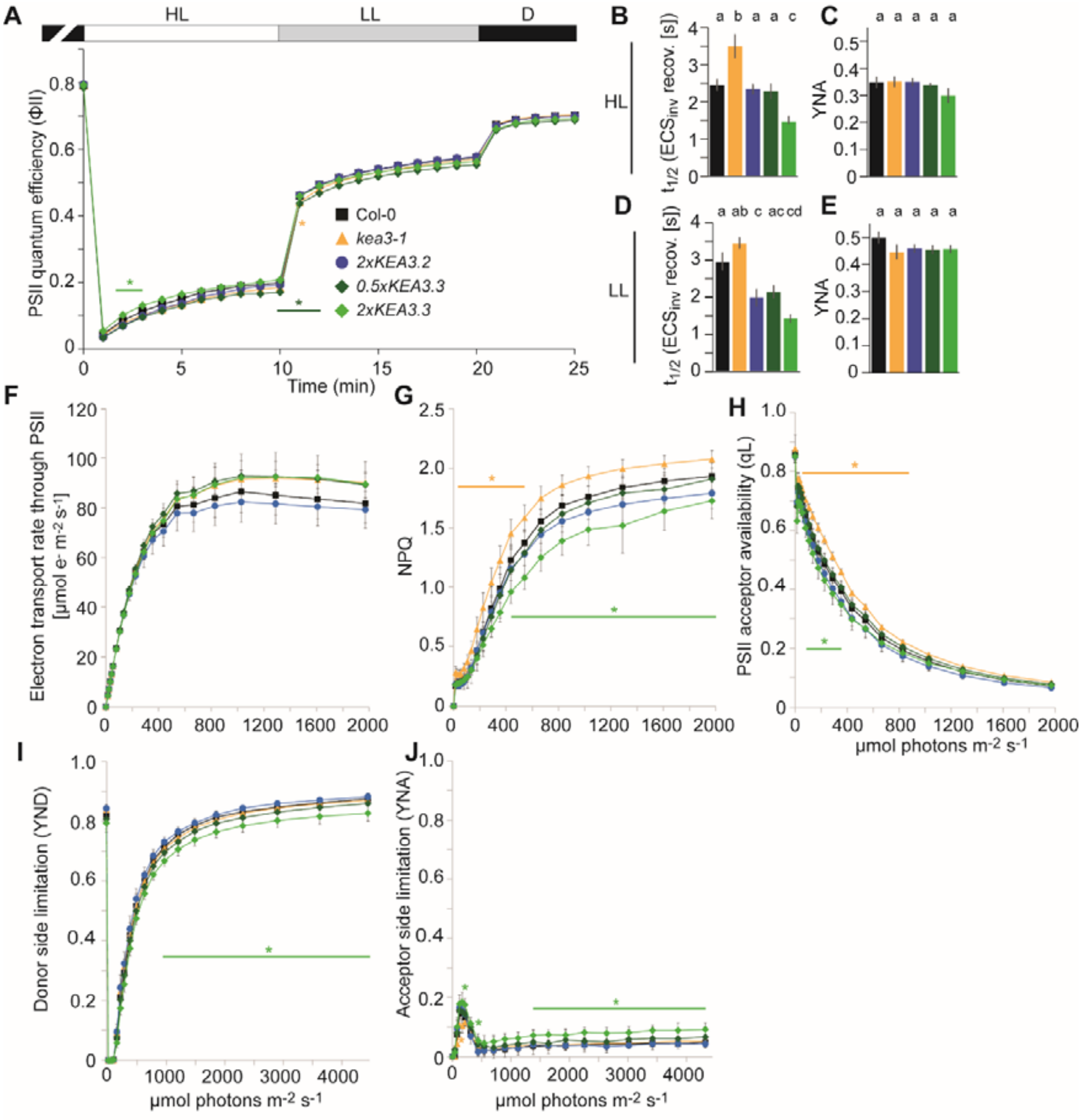

Supplemental Fig. 5

Supplemental Figure S5. *2xKEA3.3* overcompensates the photosynthetic phenotypes of *kea3-1* particularly at high light intensities.

(A) Φ_{II} calculated from the same Chl *a* fluorescence measurement as in Fig. 3B. (B-E) Halftime ($t_{1/2}$) of ECS recovery in the dark (B, D) and acceptor side limitation of PSI (YNA) after high light (B, C) and low light (D, E) as in Fig. 3. Different letters above bars indicate significant differences between genotypes with $P < 0.05$ as calculated by ANOVA and Tukey pairwise multiple comparison. (F-I) Light response curve of Chl *a* fluorescence parameters, electron transport rate through PSII (F), NPQ (G), PSII acceptor availability (qL, H) and P700 absorption parameters, PSI donor side limitation (YND, I) and PSI acceptor side limitation (YNA, J). Green and red lines with asterisks above and below the traces indicate where 2x*KEA3.3* and *kea3-1* are significantly different from WT (Col-0) as determined by ANOVA and Tukey posthoc pairwise comparison with $P< 0.05$. (B-J) Average is shown for N = 8 and error bars indicate ± SE.

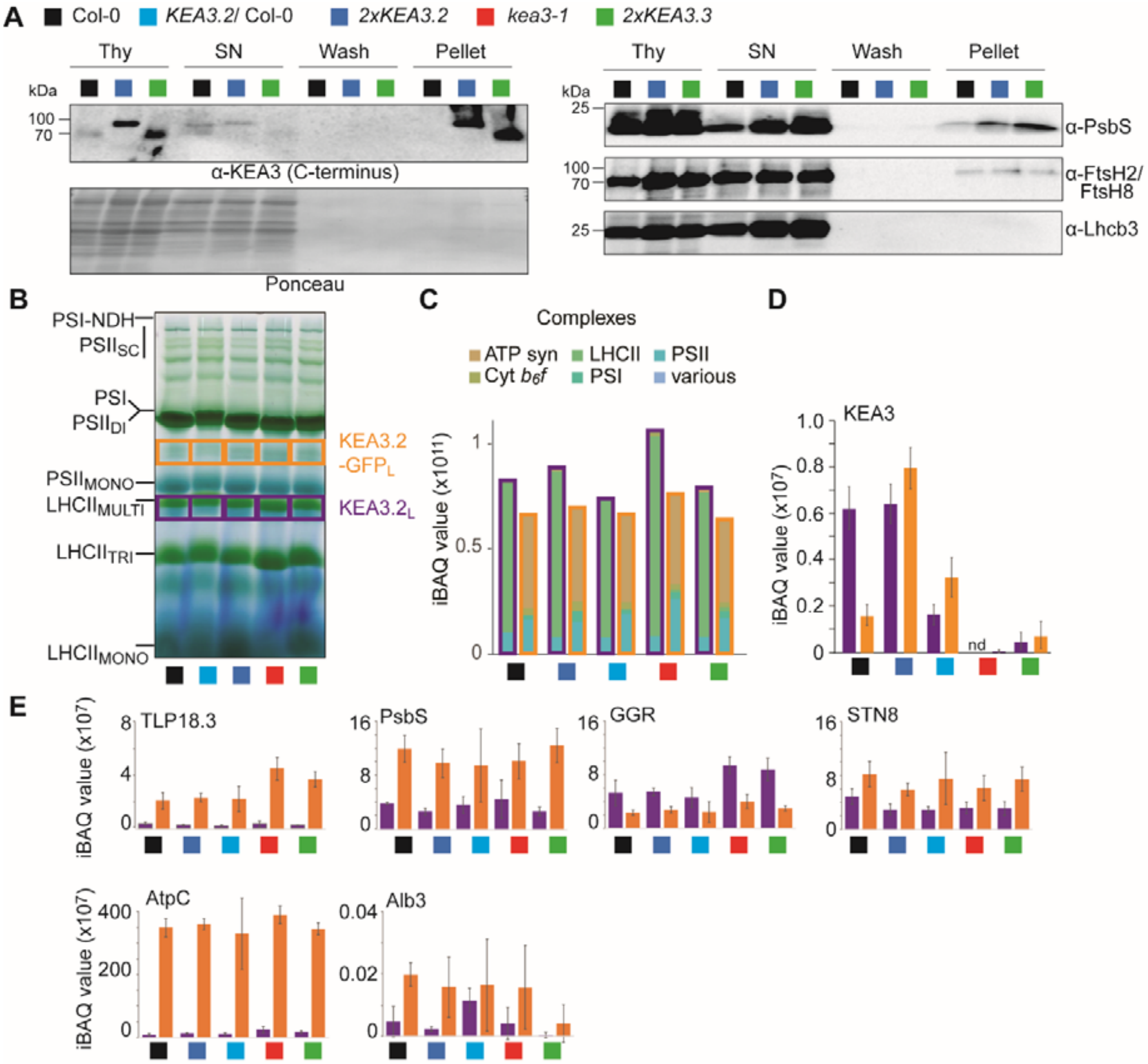

Supplemental Figure S6. No putative protein interaction partner can be confirmed for KEA3.

(A) GFP-tagged KEA3 proteins were isolated from solubilized thylakoids by using the GFP trap (Thy, thylakoids; SN, supernatant; Wash, last washing step; Pellet, extracted protein from trap) and protein blots were performed with the KEA3 C-terminal antibody and antibodies against the putative interaction partners PsbS and FtsH2/FtsH8 and the negative control Lhcb3. Ponceau stain of the membrane is shown after blotting. (B) Representative BN gel. Slices from the individual lanes were cut out at the size of KEA3.2$_L$ (purple) and KEA3.2-GFP$_L$ (orange) and proteins were analyzed by MS in triplicates. (C) Slices of the different genotypes had a similar thylakoid protein composition, supporting that slices were cut out at the same position. The KEA3.2$_L$ slices mainly contained LHCII and some PSII, while the KEA3.2-GFP$_L$ slices contained mainly ATP synthase and PSII. (D) iBAQ values of KEA3 found in the different slices. *KEA3.2-GFP/kea3-1* had a fairly high iBAQ intensity for KEA3 in the slice containing the KEA3.2$_L$ complex, while the C-terminal KEA3 antibody only gave a weak signal on immunoblots at this molecular weight (Fig. 5B). This discrepancy in results may be explainable by the detection of KEA3.2-GFP$_L$ degradation products by MS, that could not be detected by the KEA3 antibody. (E) iBAQ intensities of putative interaction partners

TLP18.3, PsbS, GGR, STN8, AtpC and Alb3 in the different slices. (D-E) Average is shown for N = 3 and error bars indicate ± SD.

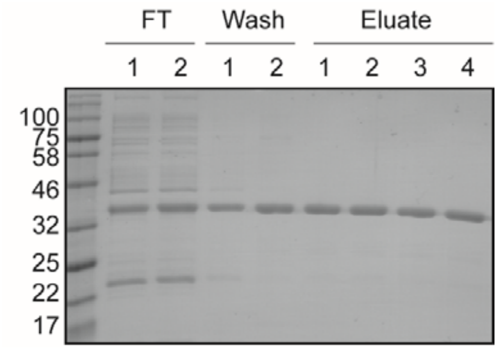

Supplemental Figure S7. Purification of recombinant KEA3 C-terminus from *E. coli*

His-tagged protein was purified by using Ni-NTA. Flow through (FT), the last washes (Wash) and eluted proteins were separated by SDS-PAGE and stained with Coomassie brilliant blue.

Supplemental Table S1: Primer names, purpose and DNA sequences used for the generation of native expression lines.

**Primer name**	**Purpose**	**Primer Sequence (5’→ 3’)**
5_UTR_fwd	Amplification 5’ UTR	*tggattttgagttaggatccgttctccggaatcaactctatc*
5_UTR_rev		*taattgccattttgagatgaaacccacaaaac*
KEA3.2/3.3_fwd	Amplification KEA3.2/KEA3.3-GFP	*tcatctcaaaatggcaattagtactatgttaggg*
KEA3.2/3.3-GFP_rev		*gaatttttgagcaattttacttgtacagctcgtcc*
KEA3-GFP 3'_UTR_fwd	Amplification 3’ UTR	*gctgtacaagtaaaattgctcaaaaattctaacac*
3_UTR_rev		*gtcttaattaactctctagaatcatgttagccgtacacgg*
